# The prognostic utility of preoperative neutrophil-to-lymphocyte ratio (NLR) in patients with colorectal liver metastasis: a systematic review and meta-analysis

**DOI:** 10.1186/s12935-023-02876-z

**Published:** 2023-02-28

**Authors:** Yanqing Li, Tianxiang Xu, Xin Wang, Xiangdong Jia, Meng Ren, Xiaoxia Wang

**Affiliations:** 1 Graduate School of Baotou Medical College, Inner Mongolia University of Science and Technology, Baotou, 014000 China; 2 Abdominal Tumor Surgery, Center of Tumor, Inner Mongolia People’s Hospital, Hohhot, 010017 China; 3 Intensive Care Unit, Inner Mongolia People’s Hospital, Hohhot, 010017 China

**Keywords:** NLR, Colorectal cancer, Liver metastasis, Prognosis, Meta-analysis

## Abstract

The neutrophil-to-lymphocyte ratio is used to reflect body's inflammatory status with prognostic value in different cancers. We aimed to investigate the influence of preoperative NLR in the prognosis of CRLM patients receiving surgery using meta-analysis. Data in Cochrane Library, PubMed, Embase, and Web of Science databases created before October 2022 were recruited. Meta-analysis was carried out with RevMan 5.3 and Stata16 software, and the primary outcome indicators included overall survival (OS), and secondary outcome indicators included disease-free survival (DFS) and relapse-free survival (RFS). The pooled risk ratio (HR) and 95% confidence interval (CI) for each outcome indicator were determined using random-effects models or fixed-effects models. The pooled odds ratio (OR) and corresponding 95% confidence intervals (CI) for NLR and clinicopathological characteristics were determined with a fixed-effects model. 18 papers published between 2008 and 2022 (3184 patients in total) were included. The pooled analysis found that high preoperative NLR was correlated with poor OS (multivariate HR = 1.83, 95% CI = 1.61–2.08, p < 0.01), DFS (multivariate HR = 1.78, 95% CI = 1.16–2.71, p < 0.01) and RFS (multivariate HR = 1.46, 95% CI = 1.15–1.85, p < 0.01), but NLR was not related to clinicopathological features of CRLM patients correlation. In conclusion, NLR is an independent risk factor for poor prognosis in patients with CRLM. More large-scale clinical researches are required in the future to demonstrate the inclusion of preoperative NLR as a prognostic indicator for CRLM patients to guide postoperative adjuvant chemotherapy.

**Supplementary Information:**

The online version contains supplementary material available at 10.1186/s12935-023-02876-z.

## Introduction

Colorectal cancer (CRC) is the third most popular malignancy [1]. In China, its incidence and mortality have shown a significant increase in the last decade [2]. Liver is the most popular site of CRC metastasis since the intestinal venous reflux enters liver through the portal vein, and around 50% of CRC patients undergo liver metastasis. Of these, 35% CRC patients only have liver metastases, and most of them eventually die from colorectal liver metastases (CRLM) [3, 4]. Hepatectomy is a potential radical treatment [5]. However, the recurrence rate is still more than 70% 5 years after surgery even with effective hepatectomy [6]. Therefore, a responsible prognostic marker is required to determine recurrence at high risk and to promote the adjuvant therapy. Several scoring systems have been established using multiple indicators, including tumor markers, pathological parameters and molecular features [7–11]. However, retrospective application of these measures with liver resection and pathological specimens is usually expensive and complicated, and scoring systems for predicting survival are specific for primarily tumor. It is well established that the host immune system exerts an important role in cancer progression. Therefore, inflammatory biomarkers are considered to be a simple, rapid and economical way to forecast the prognosis of metastatic colorectal cancer [12].

Accumulating evidence suggests that systemic inflammatory markers, originated from circulating blood leukocytes and acute phase proteins, are related to survival in a variety of tumors [13–17]. NLR is a leukocyte-based indicator of inflammation with prognostic value in cancers like colorectal cancer [15, 18]. It has been shown that the high neutrophil status in the blood leads to an increase in the secretion of vascular endothelial growth factor, playing a pro-angiogenic role in tumor development and inducing a precancerous environment [19]. In contrast, the relative lymphocytopenia leads to an inadequate response of the organism to the tumor [20]. Thus, high NLR may lead to stronger disease performance and worse outcomes. Whereas, the correlation between increased preoperative NLR and survival outcomes in CRLM patients is still controversial [12, 21]. This study aims to provide evidence-based medical evidence for the impact of preoperative NLR on the prognosis CRLM patients.

## Materials and methods

### Search strategy

A study protocol was registered with International Prospective Register of Systematic Reviews (PROSPERO), ID CRD42022326813, and reported in accordance with the PRISMA statement [22]. Cochrane Library, PubMed, Embase, and Web of Science were used. The search terms included "Colorectal Neoplasms" and its keywords, "Colonic Neoplasms" and its keywords, "Rectal Neoplasms" and its keywords, "Neoplasm Metastasis" and its keywords, "Colorectal liver metastasis" and its keywords, "Neutrophil-to-lymphocyte ratio" and its keywords. The search is conducted based on a combination of medical subject headings (MeSH) terms and keywords adjusted to the characteristics of each database. "OR" is used for the combination of MeSH terms and keywords, while "AND" is used for the combination of MeSH terms. The search strategy is shown in Additional file 2: Table S1. The last search was performed on 19 January 2022.

### Inclusion and exclusion criteria

Inclusion criteria: (1) CRLM was confirmed by pathology and all patients underwent surgical treatment; (2) the study provided preoperative NLR value as a variable in the outcome analysis; (3) studies explicitly report NLR cutoff values; (4) the hazard ratio (HR) and 95% confidence interval (CI) of the correlation between preoperative NLR and survival outcomes (overall survival (OS), disease-free survival (DFS), and recurrence-free survival (RFS)) were obtained using univariate and multivariate COX regression analysis. Overall survival (OS) was defined as the time length from operation to death for any cause, disease-free survival (DFS) was calculated from the date of hepatectomy to the date of tumor recurrence, while recurrence-free survival (RFS) was defined as the interval from hepatic resection to disease recurrence or death [23, 31].

Exclusion criteria: (1) studies related to other cancers; (2) articles, letters, case reports, commentaries or reviews, conference abstracts, research reports, reviews, and meta-analyses not related to this topic; (3) duplicate publications; (4) the full text was not available (we obtained the full text of literatures through the database provided on the school's official website VPN, the database we purchased, interlibrary loan and email authors); (5) the study with incomplete data and whose authors could not be contacted; (6) study of extrahepatic metastases in patients with CRLM; (7) study about patients treated with non-surgical treatment (chemotherapy alone); (8) non-English studies.

### Literature screening, quality evaluation and data extraction

The references were rigorously screened by 2 researchers and evaluated by another person in case of disagreement, and the decision about inclusion of the literature was made through discussion. Their quality was determined with the Newcastle-Ottawa Scale (NOS), including patient selection, group comparability, and outcome analysis. Studies of 6–9 were defined as high quality, and those ≤ 5 were seen as low quality. Extracted data included first author, publication year, research area, time of recruitment, patients number, number of patients with increased NLR, patient gender, patient age, NLR cut-off value, postoperative treatment, follow-up time, HR and corresponding 95% CI for the correlation between high preoperative NLR and survival outcomes (OS, DFS, RFS) obtained by univariate and multivariate COX regression analysis. If HR, 95% CI, or other critical data were missing, the corresponding author would be contacted. If inaccessible, the approximate estimate of HR and the corresponding 95% CI were obtained from additional information (Kaplan-Meier curves corresponding to survival outcomes) with Tierney’s method [23].

### Definition of endings

To assess the effect of preoperative neutrophil-to-lymphocyte ratio on the prognosis undergoing radical surgery, the primary outcome indicators for Meta-analysis included OS, and secondary outcome indicators included DFS and RFS.

### Statistical analysis

Meta-analysis was carried out through RevMan 5.3 software, and HR and their 95% CI were adopted to analyze the correlation between NLR and the prognosis of CRLM patients. For the correlation between NLR and clinical case traits, odds ratio (OR) and its 95% CI were adopted for assessment. Statistical heterogeneity between trials was assessed by a Chi-squared test in the absence of significant heterogeneity (I2 ≤ 50% and P ≥ 0.1), a fixed-effects model; otherwise, a random-effects model was used. Heterogeneity assessment was discussed by applying sensitivity analysis and subgroup analysis. Stata 16.0 statistical software was adopted to conduct Egger test with *P* < 0.05 as significant difference. The trim-and-fill analysis was applied to determine the groups with publication bias to determine the stability and reliability of their Meta-analysis.

## Results

### Literature search

**Fig. 1:**
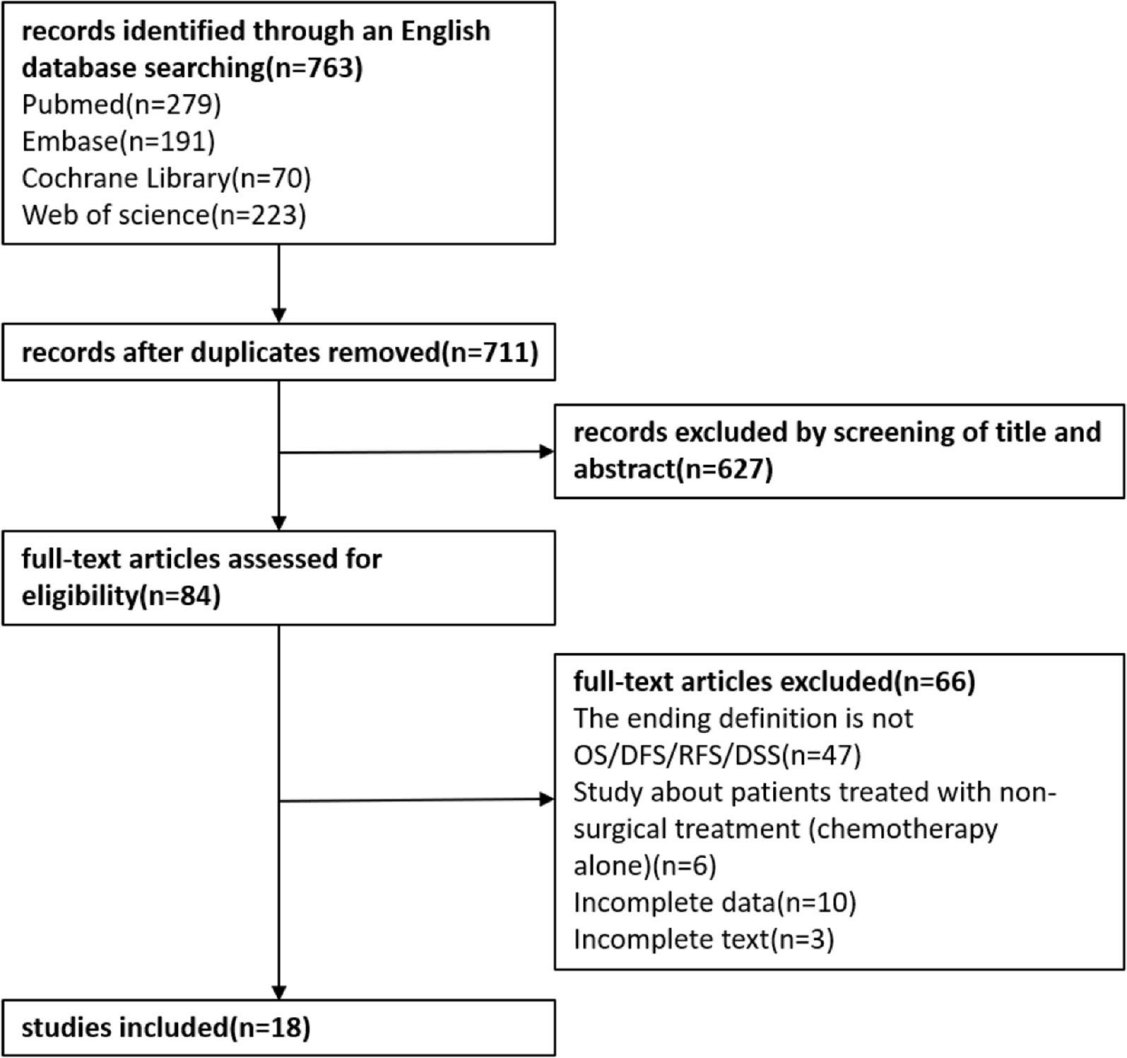
Flow chart of paper inclusion

The flow chart of the literature search is shown as Fig. 1. A total of 763 articles were retrieved. After eliminating duplicate items, 52 literatures were excluded. Another 627 were further excluded in title/abstract review. The full text of 84 literatures was then downloaded to determine their eligibility, of which 66 were excluded, including those with non-OS/DFS/RFS as an outcome indicator (n = 47), those with not relevant topic (n = 6), those with incomplete data (n = 10), and those for which the full text could not being downloaded (n = 3). Ultimately, 18 articles (3184 patients) published between 2008 and 2021 were included in this meta-analysis [3, 12, 21, 24–38]. All studies were retrospective cohort studies in which 2857 patients underwent hepatic resection, 92 patients underwent percutaneous radiofrequency ablation, and 235 patients underwent radioembolization. The characteristics of the included literature are summarized in Table 1. Six researches were conducted in the United Kingdom, five in China, three in the United States, one in Japan, one in Korea, one in Poland, and one in Israel. The NLR cutoff value of 10 articles was ≠ 5, and 8 articles was 5. All studies were considered high quality according to the NOS score, ranging from 7 to 8 (Table 2).

**Table 1:** The key traits and the quality evaluation of the included researches

Study (Year)	Country	Time	N	High NLR	M/F	Age(year)	NLR cut-off	Median survival(year)				Postoperative chemotherapy	Follow-up (months)	Outcome	NOS
Verter (2021) [12]	Israel	2005–2017	231	53	131/100	66.3a	3	NLR ≤ 3 NLR > 3	OS 5.2 3.8	DSS 6.5 4.2	RFS 1.2 0.8	Neoadjuvant chemotherapy (n = 175) Adjuvant chemotherapy (n = 166)	180	OS/DSS/RFS	8
Wang (2019) [24]	China	2002–2016	452	–	289/163	57b	2.6	–				Postoperative chemotherapy (n = 284)	84	OS/DFS	8
Kim (2019) [3]	Korea	2005–2015	83	41	62/21	59.5a	1.94	NLR < 1.94 NLR ≥ 1.94	OS 6.5 3.4	DFS 2.5 1.1		5-FU plus LV (n = 6) FOLFOX (n = 32) FOLFOX plus Beva (n = 2) FOLFIRI (n = 30) FOLFIRI plus Beva (n = 8) FOLFIRI plus Cetu (n = 5)	140	OS/DFS	7
Neal (2015) [25]	UK	2006–2010	302	53	192/110	59.5a	3	NLR < 5 NLR ≥ 5	OS 3.3 2.3	CSS 3.8 2.3		Neoadjuvant chemotherapy (n = 126)	96	OS/DFS	8
Neal (2011) [26]	UK	2000–2006	202	28	126/76	61.5a	5	–				Neoadjuvant chemotherapy (n = 84)	70	OS/DFS	7
Neal (2009) [27]	UK	2000–2005	174	21	106/68	65b	5	–				Adjuvant chemotherapy (n = 67)	69	OS/DFS	7
Mao (2019) [28]	China	2006–2015	183	104	123/60	60b	2.3	NLR ≤ 2.3 NLR > 2.3	OS 3.6 2.6	RFS 0.9 0.5		Postoperative chemotherapy (n = 143)	69	OS/RFS	8
Halazun (2008) [29]	UK	1996–2006	440	78	289/151	64a	5	NLR < 5 NLR ≥ 5	OS 4.5 2.2	DFS 2.9 1.1		5-FU plus CF (n = 440)	97	OS	8
Erstad (2020) [30]	US	1995–2017	151	40	84/67	64a	5	NLR < 5 NLR ≥ 5	OS 6.3 3.1			–	186	OS	7
Neofytou (2014) [31]	UK	2005–2012	140	63	88/52	≤ 70 (n = 109) > 70 (n = 31)	2.4	NLR ≤ 2.4 NLR > 2.4	OS c 4.6	DFS 1.4 0.9		neoadjuvant chemotherapy (n = 140)	103	OS/DFS	7
Peng (2017) [32]	China	2000–2012	150	16	97/53	58b	4.63	NLR < 4.63 NLR ≥ 4.63	OS 3.7 1.5	RFS 1.9 0.7		postoperative chemotherapy (n = 110)	126	OS/RFS	7
Zeman (2013) [33]	Poland	2001–2009	96	–	–	–	5	NLR ≤ 5 NLR > 5	DFS 1.8 1			5-FU plus LV (n = 20) FOLFOX (n = 5) Adjuvant chemotherapy (n = 25)	156	DFS	8
Giakoustidis (2015) [34]	UK	2005–2012	169	71	104/65	≤ 70 (n = 135) > 70 (n = 34)	2.5	NLR ≤ 2.4 NLR > 2.4	OS c 6.3			Oxaliplatin-based chemotherapy (n = 111) Irinotecan- based chemotherapy (n = 52) Other (n = 6)	101	OS/DFS	7
Wu (2016) [35]	China	2008–2013	55	14	35/20	59b	4	NLR < 4 NLR ≥ 4	OS c 1.5	PFS 1 0.6		mFOLFOX6 (n = 21) XELOX (n = 34)	80	OS/PFS	7
Hamada (2020) [21]	Japan	2000–2008	29	3	20/9	63	4.1	–				mFOLFOX6 (n = 21) XELOX (n = 34)	97	OS	7
Zhang (2012) [36]	China	2000–2008	92	21	61/41	≤ 65 (n = 48) > 65 (n = 44)	5	NLR ≤ 5 NLR > 5	OS 3.5 2.2	DFS 2.1 0.9		–	70	OS/DFS	7
Weiner (2018) [37]	US	–	131	–	84/47	–	5	NLR < 5 NLR ≥ 5	OS 1.1 0.7			–	–	OS	7
Tohme (2014) [38]	US	2002–2012	104	48	–	< 70 (n = 61) ≥ 70 (n = 43)	5	NLR < 5 NLR ≥ 5	OS 1.1 0.7			–	70	OS	7

M: male; F: female; a: mean age; b: median age; c: median OS was not reached in patients; 5-FU: 5-fluorouracil; LV: leucovorin; FOLFOX: 5-fluorouracil, leucovorin and oxaliplatin; Beva: bevacizumab; FOLFIRI: 5-fluorouracil, leucovorin and Irinotecan; Cetu: cetuximab; CF: folinic acid; mFOLFOX6: folic acid/fluorouracil plus oxaliplatin; XELOX: capecitabine plus oxaliplatin; CSS: Cancer-specific survival; PFS: Progression-free survival. “—”: no data

**Table 2:** NOS scale quality assessment

Author (Year)	Selection	Comparability	Outcomes	Quality score
Verter (2021) [12]	★★★	★★★	★★★	8
Wang (2019) [24]	★★★	★★★	★★★	8
Kim (2019) [3]	★★★	★★★	★★★	7
Neal (2015) [25]	★★★	★★★	★★★	8
Neal (2011) [26]	★★★	★★★	★★★	7
Neal (2009) [27]	★★★	★★★	★★★	7
Mao (2019) [28]	★★★	★★★	★★★	8
Halazun (2008) [29]	★★★	★★★	★★★	8
Erstad (2020) [30]	★★★	★★★	**★★★**	7
Neofytou (2014) [31]	★★★	★★★	★★★	7
Peng (2017) [32]	★★★	★★★	★★★	7
Zeman (2013) [33]	★★★	★★★	★★★	8
Giakoustidis (2015) [34]	★★★	★★★	★★★	7
Wu (2016) [35]	★★★	★★★	★★★	7
Hamada (2020) [21]	★★★	★★★	★★★	7
Zhang (2012) [36]	★★★	★★★	★★★	7
Weiner (2018) [37]	★★★	★★★	★★★	7
Tohme (2015) [38]	★★★	★★★	★★★	7

★: 1 point

### Literature search

**Fig. 2:**
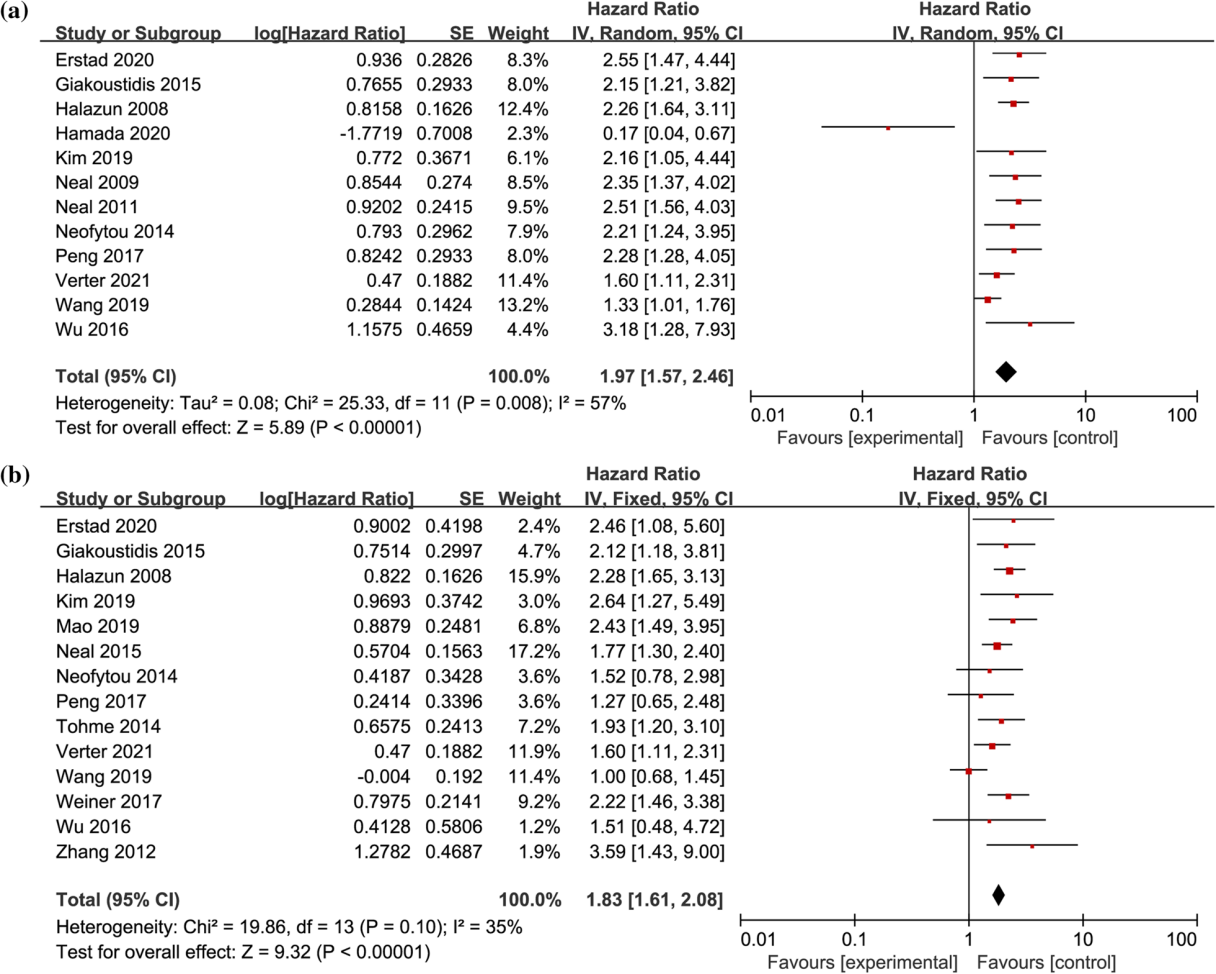
Forest plot of the correlation between preoperative NLR and OS in CRLM patients. **a** univariate OS; **b** multivariate OS; OS, overall survival; NLR, Neutrophil-to-Lymphocyte

Twelve studies provided HR and 95% CI for preoperative NLR on univariate OS in CRLM patients [3, 12, 21, 24, 26, 27, 29–32, 34, 35], including 2276 patients in total. Heterogeneity test showed that there was statistical heterogeneity among studies (I^2^ = 57%, *P* < 0.01). Meta-analysis with random-effects model demonstrated (Fig. 2a) that the pooled HR = 1.97, 95% CI = 1.57–2.46, *P* < 0.01. Fourteen studies provided HR and 95% CI for multifactorial OS in patients with CRLM [3, 12, 24, 25, 28–32, 34–38], including 2683 patients in total. Heterogeneity test results suggested no statistical heterogeneity among studies (I^2^ = 35%, *P* = 0.1). Meta-analysis with fixed-effects model demonstrated (Fig. 2b) the pooled HR = 1.83, 95% CI = 1.61–2.08,* P* < 0.01, showing that patients with high preoperative NLR had poor OS.

### Association of preoperative NLR with univariate and multivariate DFS in CRLM patients

**Fig. 3:**
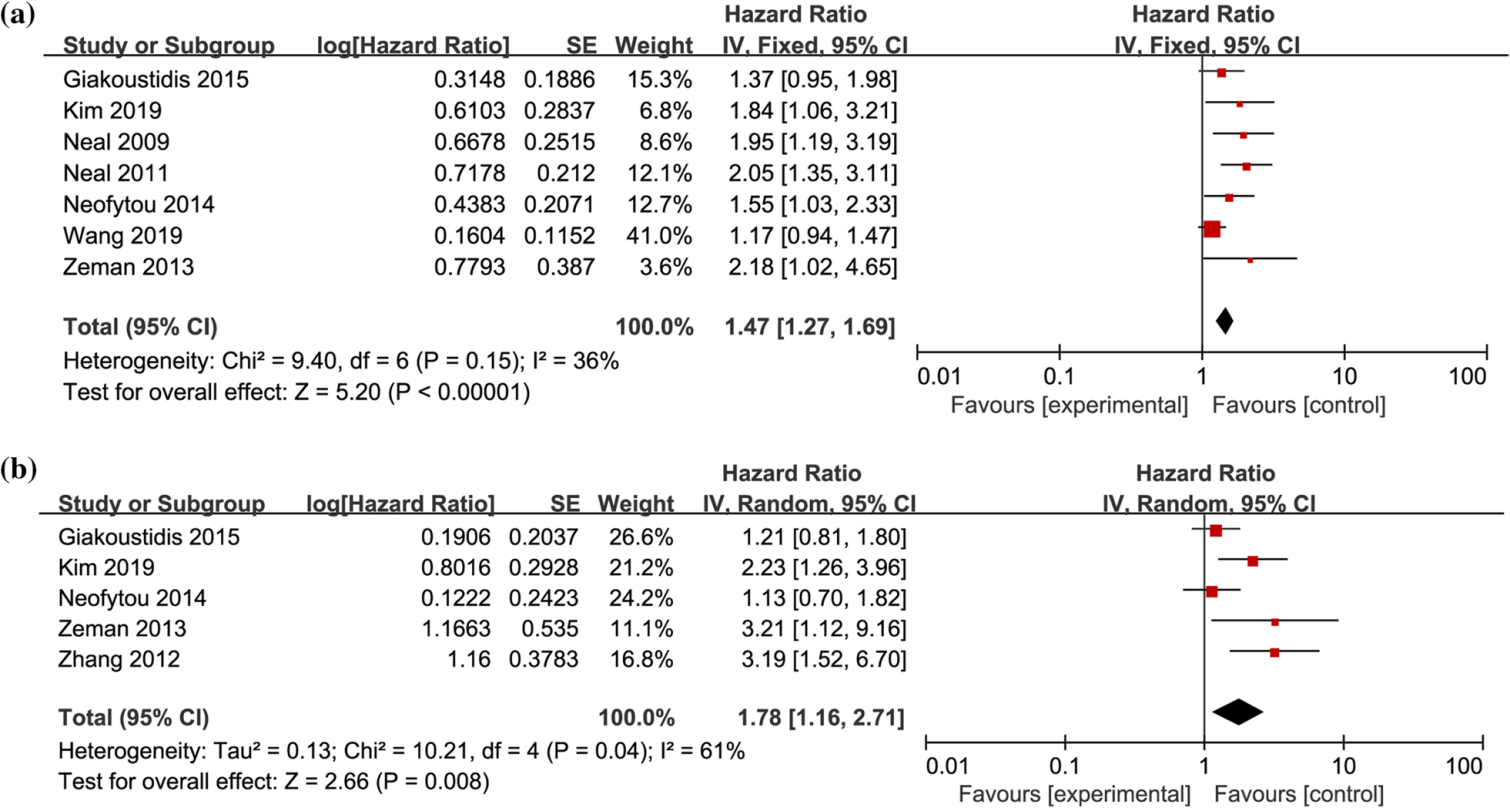
Forest plot of the relationship between preoperative NLR and DFS in CRLM patients. **a** univariate DFS; **b** multivariate DFS; DFS, disease-free survival; NLR, Neutrophil-to-Lymphocyte

Seven studies provided HR and 95% CI for preoperative NLR on univariate DFS in CRLM patients [3, 24, 26, 27, 31, 33, 34], including 1316 patients in total. Heterogeneity test found no significant heterogeneity among the studies (I^2^ = 36%, *P* = 0.15). Meta-analysis with fixed-effects model demonstrated (Fig. 3a) that the pooled HR = 1.47, 95% CI = 1.27–1.69, *P* < 0.01. Five researches provided HR and 95% CI for multifactorial DFS in patients with CRLM [3, 31, 33, 34, 36], including 580 patients in total. Heterogeneity test found statistical heterogeneity among studies (I^2^ = 61%, *P* = 0.04). Meta-analysis with random-effects model showed (Fig. 3b) the pooled HR = 1.78, 95% CI = 1.16–2.71, *P* < 0.01, suggesting that patients with high preoperative NLR were related to poorer DFS.

### Correlation of preoperative NLR with univariate and multivariate RFS in patients with CRLM

**Fig. 4:**
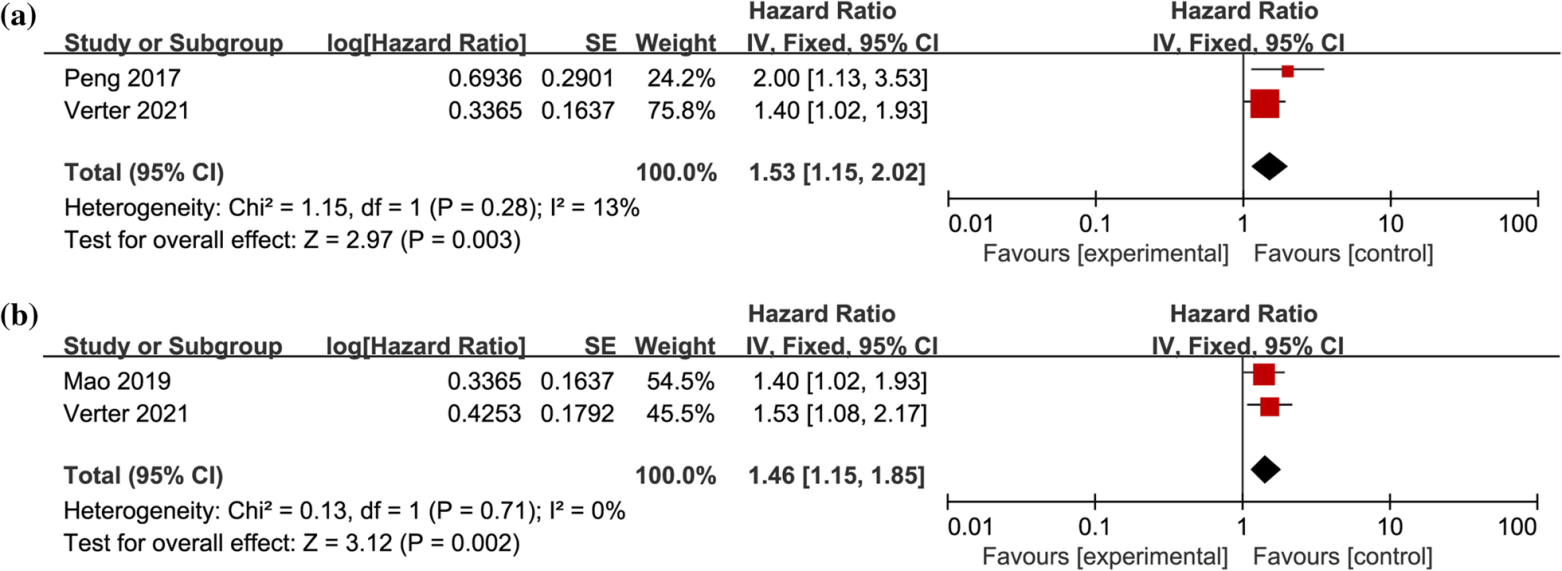
Forest plot of the association between preoperative NLR and RFS in CRLM patients. **a** univariate RFS; **b** multivariate RFS; RFS, recurrence-free survival; NLR, Neutrophil-to-Lymphocyte

Two studies provided HR and 95% CI for preoperative NLR on univariate RFS in CRLM patients [12, 32], including 381 patients in total. Heterogeneity test results suggested no statistical heterogeneity among studies (I^2^ = 13%, *P* = 0.28). Meta-analysis with fixed-effects model demonstrated indicated (Fig. 4a) that the pooled HR = 1.53, 95% CI = 1.15–2.02, *P* < 0.01. Two researches provided HR and 95% CI for multifactorial RFS in patients with CRLM [12, 28], including 414 patients in total. Heterogeneity test results suggested no statistical heterogeneity among studies (I^2^ = 0%, *P* = 0.71), with HR = 1.46, 95% CI = 1.15–1.85, *P* < 0.01, suggesting that patients with high preoperative NLR were related to poorer RFS (Fig. 4b).

### Correlation of preoperative NLR with clinicopathologic characteristics in CRLM patients

Eight studies provided OR and 95% CI for the number of metastases in patients with preoperative NLR versus CRLM [3, 12, 28, 29, 31, 32, 34, 36], including 1488 patients in total. The heterogeneity test found no statistical heterogeneity among studies (I^2^ = 10%, *P* = 0.35). Meta-analysis with fixed-effects model demonstrated a pooled OR = 1, 95% CI = 0.76–1.31, *P* = 0.97 without statistical significance (Table 3, Additional file 1: Fig. S1a).

**Table 3:** Meta-analysis results of the correlation between preoperative NLR and clinicopathological features in patients with CRLM

Clinical case characteristics	Number of Studies	Heterogeneity test results		Effect model	Meta-analysis results	
		I^2^ (%)	P		OR (95% CI)	P
Number of metastases	8	10	0.35	Fixed-effects model	1 (0.76–1.31)	P = 0.97
CEA	6	46	0.1	Fixed-effects model	1.31 (0.88–1.96)	P = 0.19
Primary tumor size	6	0	0.89	Fixed-effects model	1.22 (0.90–1.67)	P = 0.2
Time to metastasis	4	0	0.89	Fixed-effects model	1.12 (0.78–1.62)	P = 0.54

Number of metastases, > 3 or < 3; CEA, > CEA cut-off or < CEA cut-off; primary tumor size, > 5 cm or < 5 cm; time to metastasis, Synchronous transfer or Metachronous transfer

Six studies provided OR and 95% CI for preoperative carcinoembryonic antigen (CEA) level in patients with NLR versus CRLM [3, 12, 32, 34, 36, 38], including 829 patients in total. The heterogeneity test found no statistical heterogeneity among studies (I^2^ = 46%, *P* = 0.1). Meta-analysis with fixed-effects model demonstrated that the pooled with OR = 1.31, 95% CI = 0.88–1.96, *P* = 0.19, had no statistical significance (Table 3, Additional file 1: Fig. S1b).

Six studies provided OR and 95% CI for primary tumor size in patients with preoperative NLR versus CRLM [3, 12, 29, 31, 32, 36], including 1236 patients in total. The heterogeneity test found no statistical heterogeneity between studies (I^2^ = 0%, *P* = 0.89), with OR = 1.22, 95% CI = 0.90–1.67, *P* = 0.2 (Table 3, Additional file 1: Fig. S1c).

Four studies provided OR and 95% CI for time to metastasis in patients with preoperative NLR versus CRLM [12, 31, 32, 34], including690 patients in total. The heterogeneity test found no statistical heterogeneity between studies (I^2^ = 0%, *P* = 0.86), with a pooled OR = 1.12, 95% CI = 0.78–1.62, *P* = 0.54 (Table 3, Additional file 1: Fig. S1d).

### Sensitivity analysis and subgroup analysis

**Fig. 5:**
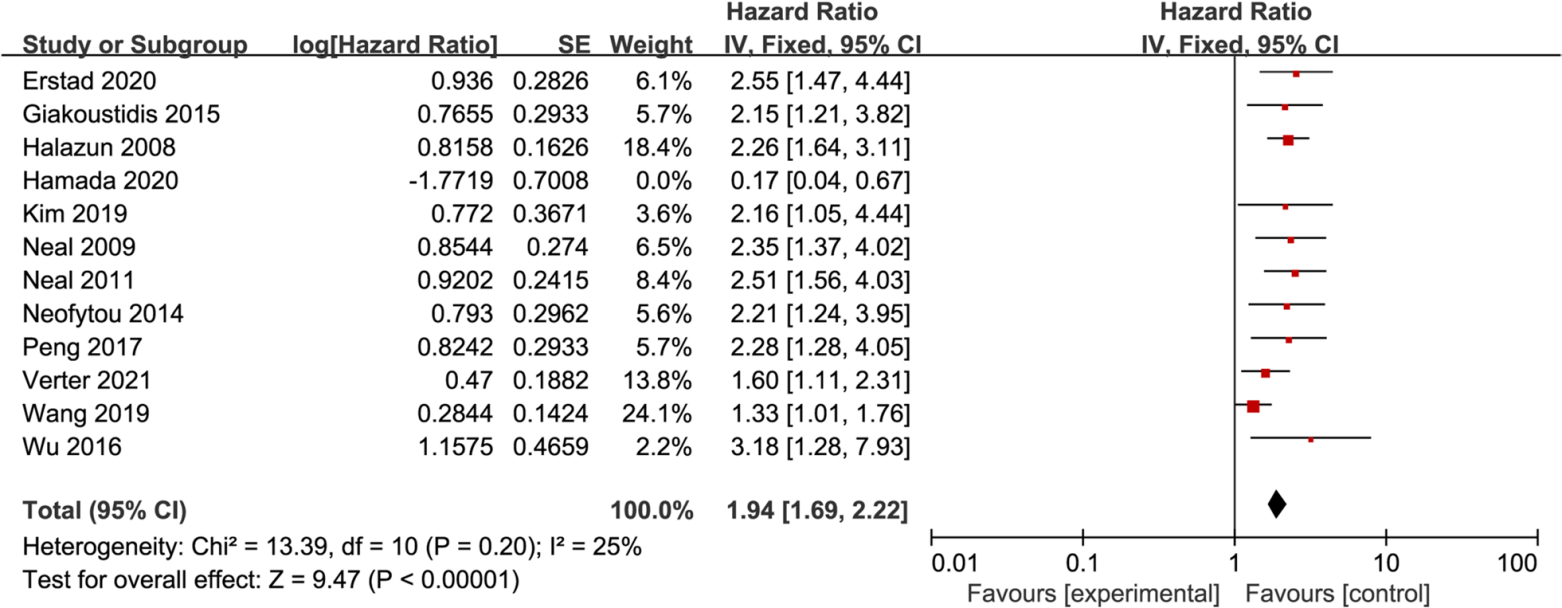
Forest plot for sensitivity assessment of the relationship between NLR and univariate OS in CRLM patients

Heterogeneity test results showed statistical heterogeneity in univariate OS and multivariate DFS of preoperative NLR in CRLM patients (I^2^ = 57%, *P* < 0.01; I^2^ = 52%, *P* = 0.1). As shown in the univariate OS dendrogram (Fig. 2a), study deviations such as Hamada et al. are obvious and may be the main source of heterogeneity. Using sensitivity analysis (deleting a single study), the heterogeneity was pronouncedly inhibited by excluding the research of Hamada et al. (I^2^ = 25%, *P* = 0.2). The Meta-analysis with fixed-effects model demonstrated (Fig. 5) the pooled HR = 1.94, 95% CI = 1.69–2.22, *P* < 0.01, without significant differences, indicating results stable and reliable.

A subgroup analysis of the multivariate DFS was performed, with groupings based on whether the sample size (N) of the study was  ≥ 100. N < 100 group (I^2^ = 0%, *P* = 0.55), Meta-analysis with fixed-effects model showed (Table 4, Additional file 1: Fig. S2a) combined effect size HR = 2.64, 95% CI = 1.71–4.01, *P* < 0.01. N ≥ 100 group (I^2^ = 0%, P = 0.83), with HR = 1.18, 95%CI = 0.87–1.60, *P* = 0.3.

**Table 4:** Results for subgroup assessment of the relationship between NLR and multivariate DFS in CRLM patients

Subgroup	Number of studies	Heterogeneity test results		Effect model	Meta-analysis results	
		I^2^ (%)	*P*		HR (95% CI)	*P*
N < 100	3	0	0.70	Fixed-effects model	2.64 (0.76,1.31)	*P* < 0.01
N ≥ 100	2	0	0.83	Fixed-effects model	1.18 (0.87,1.60)	*P* = 0.3
NLR cut-off value = 5	2	5	2	Fixed-effects model	3.20 (1.74,5.86)	*P* < 0.01
NLR cut-off value < 5	3	5	3	Fixed-effects model	1.35 (1.03,1.77)	*P* = 0.03

Patients were classed into two groups based on the study NLR cutoff value. NLR cut-off value = 5 group (I^2^ = 0%, *P* = 0.99), Meta-analysis with fixed-effects model (Table 3) combined effect size HR = 3.20, 95% CI = 1.74–5.86, *P* < 0.01.In groups with NLR cut-off value below 5 (I^2^ = 47%, P = 0.15), Meta-analysis with fixed-effects model found (Table 4, Additional file 1: Fig. S2b) combined effect size HR = 1.35, 95%CI = 1.03–1.77, *P* = 0.03.

### Publication bias

Since none of the correlation results of preoperative NLR on clinical case characteristics were statistically significant, the Egger test was not performed. For studies with t more than 2 literatures, the Egger test was recruited to analyze publication bias (Additional file 1: Fig. S3a–d), which revealed publication bias only for the univariate DFS study of preoperative NLR in CRLM patients with *P* = 0.009.* P* values of the preoperative NLR for univariate OS, multivariate OS, and multivariate DFS studies in CRLM patients were 0.852, 0.492, and 0.057, respectively (*P* > 0.05) without publication bias. The pooled HR = 1.281, 95% CI = 1.031–1.531 after applying the trim-and-fill (Additional file 1: Fig. S3e) to the univariate DFS study with unchanged significance, indicating reliable and stable results.

## Discussion

Forecasted NLR value of various tumors is confirmed. Several meta-analyses found that high NLR are related to poorer prognosis in esophageal [39, 40], gastric [41–43], hepatocellular [44–46], cholangiocarcinoma [47], colorectal [48–50], non-small cell lung [51, 52], breast [53], ovarian [54], renal cell [55, 56], and prostate cancers [57]. However, Hamada et al. revealed that low preoperative NLR was related to impaired univariate OS in CRLM patients, while multiple studies reported that high preoperative NLR was a symbol of poor CRLM prognosis. Thus, it can be seen that there is contradictory about prognostic significance of preoperative NLR in CRLM. In a meta-analysis of 3184 CRLM patients from 18 studies, high preoperative NLR was correlated with OS (univariate HR = 1.97, 95% CI = 1.57–2.46, *p* < 0.01; multifactorial HR = 1.83, 95% CI = 1.61–2.08, *P* < 0.01), DFS (univariate HR = 1.47; 95% CI = 1.27–1.96; *P* < 0.01; multifactorial HR = 1.78, 95% CI = 1.16–2.71, *P* < 0.01) and RFS (univariate HR = 1.53, 95% CI = 1.15–2.02, *P* < 0.01; multifactorial HR = 1.46, 95% CI = 1.15–1.85, *P* < 0.01) were impaired. In summary, high preoperative NLR is an independent risk factor.

According to Meta-analysis results, no correlation was found in preoperative NLR on tumor clinicopathological characteristics, including the number of metastases (OR = 1, 95% CI = 0.76–1.31, *P* = 0.97), CEA (OR = 1.31, 95% CI = 0.88–1.96, *P* = 0.19), primary tumor size (OR = 1.22, 95% CI = 0.90–1.67, *P* = 0.2) and time to metastasis (OR = 1.12, 95% CI = 0.78–1.62, *P* = 0.54)). Interestingly, Kim3 et al.’s study showed that poorly differentiated colorectal cancer and high CEA levels (cut-off 100 ng/mL) between groups were significantly associated with high NLR. Peng11 et al. found that patients with high preoperative NLR were more likely to promote multiple liver metastases than those with low preoperative NLR. Zang1 et al. found that patients with increased NLR are more likely to own relative lymphopenia and neutrophilia. Tohme et al.’s showed that patients with high NLR owned a higher incidence of extrahepatic disease when undergoing radioembolization. Overall, more prospective studies are needed to discuss the clinicopathological correlation between NLR and tumors.

Statistical heterogeneity was found in univariate OS and multifactorial DFS in CRLM patients by preoperative NLR (I^2^ = 57%, *P* = 0.008; I^2^ = 52%, *P* = 0.1) using the heterogeneity test. The heterogeneity was significantly reduced by sensitivity analysis (deleting a single study), excluding Hamada et al.’s study (I^2^ = 25%, *P* = 0.2), and the Meta-analysis results were not significantly altered. As this study was the only one with negative results included in this group, among the 29 patients included, only 3 patients had high NLR. Small sample size may be the main reason for its heterogeneity. Subgroup analysis of the multifactorial DFS was performed, and studies were grouped according to whether they owned a sample size (N)  ≥ 100. Small sample (N < 100) researches did not differ significantly from the pooled results (HR = 2.64, 95% CI = 1.71–4.01, *P* < 0.01). However, the multifactorial DFS relationship between preoperative NLR and CRLM patients was significantly lower in studies with large samples (N ≥ 100) without statistical significance (HR = 1.18, 95% CI = 0.87–1.60, *P* = 0.3). The results of Meta-analysis with grouping were obtained based on NLR cutoff values, studies with NLR cutoff values equaling 5 had a more significant preoperative NLR correlation with multifactorial DFS in CRLM patients (HR = 3.20, 95% CI = 1.74–5.86, *P* < 0.01). In contrast, studies with NLR cutoff values below 5 had significantly lower correlations with multifactorial DFS (HR = 1.35, 95% CI = 1.03–1.77, *P* < 0.01). Therefore, the choice of NLR cutoff value remain worthy for further study and discussion.

The reasons for the correlation of increased preoperative NLR with poor survival outcome in CRLM patients are sophisticated and hard to elucidated, and the following explanations may account for this association. Cell groups such as tumor cells, normal tissue cells, mesenchymal cells, and immune cells constitute the tumor microenvironment and are closely associated with tumor development [58–60], while immune cells exert different roles in the immune response process of tumors [61, 62].

Tumor hypoxia, necrosis, and associated anti-apoptotic signaling pathways activate systemic inflammation in malignant tumors [63]. Granulocyte colony stimulating factor (GCSF) released by tumors works particularly on bone marrow granulocytes, thereby triggering neutrophilia [64]. In turn, neutrophilia promotes the secretion of vascular endothelial growth factor (VEGF), matrix metalloproteinase 9 (MMP-9), which in turn accelerates tumor development [65, 66]. VEGF is one of the most biologically active cytokines among the pro-angiogenic factors, especially acting on tumor-nourishing neovascularization [67]. Matrix metalloproteinase 9 (MMP-9) leads to the degradation of the extracellular matrix with connective tissue remodeling in the internal environment [68, 69]. MMP-9 activates VEGF and fibroblast growth factor-2 (FGF-2), which promotes vascular endothelial cell proliferation, pro-angiogenesis and signaling, providing nutrition to tumors and promoting tumor metastasis [70]. Neutrophil elastase (NE) is mainly derived from neutrophils. When the organism is in a state of tumor cell invasion, neutrophils rapidly release high levels of NE to kill tumors [71, 72]. Interestingly, Houghton et al. reported that NE has a tumor growth-promoting impact against lung tumor [73]. It was also shown that moderate concentrations of NE directly induced proliferation of tumor cells, whereas excess Ne led to tumor cell death, which emphasizes the importance of the active state of neutrophils on the biological behavior of tumors. Recent evidence has identified novel functions of neutrophils, polarizing into different phenotypes to react to environmental signals in the tumor microenvironment (anti-tumor M1 and pro-tumor M2 phenotype) [74]. M1 neutrophils enhance the body's anti-tumor function and improve host immunity by producing the tumor necrosis factor-α (TNF-α), etc., and reducing arginase level. In another hand, M2 neutrophils facilitate tumor growth through expressing arginase, MMP-9, VEGF and multiple chemokines [75, 76]. This part of neutrophils infiltrates in tumor tissue and has pronounced effects in promoting tumor proliferation, invasion, angiogenesis and metastasis. Therefore, elevated NLR may suggest a higher level of M2 phenotype neutrophil infiltration.

For a long time, it was believed that the anti-tumor activity of the organism is primarily a cellular immune response mediated by lymphocytes. Lymphocytes, as key components of the innate and acquired immunity, eradicate tumor cells by inducing cytotoxic death and secreting cytokines [77]. Fuchs et al. found that good long-term survival in CRC patients was closely associated with elevated tumor-infiltrating lymphocytes (TILs) [78], and the decrease in lymphocyte count symbolized the depressed immune defense against tumors [79]. Besides, enhanced neutrophils in peripheral blood suppressed the killing activity of lymphocytes and NK cells against tumor cells [80]. Thus, elevated NLR caused by neutrophilia or lymphocytopenia signifies the promising inhibition of host immune surveillance and response to malignancy.

This is the first Meta-analysis on the relevance of preoperative NLR on survival outcomes in CRLM patients with some limitations: (1) Most of the included studies were retrospective, relatively affecting results’ accuracy. (2) Definition differences in NLR cutoff values (cutoff = 5 in 5 studies and cutoff ≠ 5 in 10 studies) may lead to heterogeneity and variability and affect the clinical application. (3) The reliability of the statistical results may be weakened by the small size included in the subgroup analysis. (4) Three literatures were excluded in the full text was not available, which may have affected the comprehensiveness of the included literature. Therefore, these conclusions still need to be interpreted with caution.

## Conclusions

In summary, high preoperative NLR is an independent risk factor for poor prognosis and is closely associated with poorer long-term survival (OS, DFS and RFS) in CRLM patients. Therefore, preoperative NLR can be one of the biomarkers to forecast the prognosis of CRLM patients who underwent surgical resection. On the one hand, preoperative NLR can effectively and rapidly recognize patients at high risk of recurrence, and on the other hand, physicians can further promote their adjuvant therapy. However, further multicenter prospective researches are still needed to discuss in determining the optimal preoperative NLR cut-off value.

## Supplementary Information

**Additional file 1: Figure S1.** Forest plot of the relationship between preoperative NLR and Clinicopathological features in CRLM patients (a: Clinicopathological features; b: CEA; c: primary tumor size; d: time to metastasis). **Figure S2.** Forest plot for subgroup analysis of the correlation between NLR and multivariate DFS in CRLM patients (a: sample size; b: NLR cut-off value). **Figure S3.** Publication bias (a: Egger’s publication bias plot of univariate OS; b: Egger's publication bias plot of multivariate OS; c: Egger's publication bias plot of univariate DFS; d: Egger's publication bias plot of multivariate DFS; e: Funnel plot after univariate DFS application of trim-and-fill).

**Additional file 2: Table S1.** Search strategy.

## Data Availability

All data for this article can be obtained from the corresponding author.

## References

[1] Bray F, Ferlay J, Soerjomataram I, Siegel RL, Torre LA, Jemal A (2018). Global cancer statistics 2018: GLOBOCAN estimates of incidence and mortality worldwide for 36 cancers in 185 countries. Ca-Cancer J Clin.

[2] Chen W, Sun K, Zheng R, Zeng H, Zhang S, Xia C, Yang Z, Li H, Zou X, He J (2018). Cancer incidence and mortality in China, 2014. Chinese J Cancer Res.

[3] Kim H, Jung HI, Kwon SH, Bae SH, Kim HC, Baek MJ, Lee MS (2019). Preoperative neutrophil-lymphocyte ratio and CEA is associated with poor prognosis in patients with synchronous colorectal cancer liver metastasis. Ann Surg Treat Res.

[4] Jones RP, Kokudo N, Folprecht G, Mise Y, Unno M, Malik HZ, Fenwick SW, Poston GJ (2016). Colorectal liver metastases: a critical review of state of the art. Liver Cancer.

[5] Tie J, Wang Y, Cohen J, Li L, Hong W, Christie M, Wong HL, Kosmider S, Wong R, Thomson B (2021). Circulating tumor DNA dynamics and recurrence risk in patients undergoing curative intent resection of colorectal cancer liver metastases: a prospective cohort study. Plos Med.

[6] Hallet J, Sa CA, Adam R, Goere D, Bachellier P, Azoulay D, Ayav A, Gregoire E, Navarro F, Pessaux P (2016). Factors influencing recurrence following initial hepatectomy for colorectal liver metastases. Brit J Surg.

[7] Wachtel H, Hutchens T, Baraban E, Schwartz LE, Montone K, Baloch Z, LiVolsi V, Krumeich L, Fraker DL, Nathanson KL (2020). Predicting metastatic potential in pheochromocytoma and paraganglioma: a comparison of PASS and GAPP scoring systems. J Clin Endocr Metab.

[8] Nakagawa S, Hayashi H, Nitta H, Okabe H, Sakamoto K, Higashi T, Kuroki H, Imai K, Hashimoto D, Sakamoto Y (2015). Scoring system based on tumor markers and Child-Pugh classification for HCC patients who underwent liver resection. Anticancer Res.

[9] Barbon C, Margonis GA, Andreatos N, Rezaee N, Sasaki K, Buettner S, Damaskos C, Pawlik TM, He J, Wolfgang CL (2018). Colorectal liver metastases: does the future of precision medicine lie in genetic testing?. J Gastrointest Surg.

[10] Ruppert AS, Dixon JG, Salles G, Wall A, Cunningham D, Poeschel V, Haioun C, Tilly H, Ghesquieres H, Ziepert M (2020). International prognostic indices in diffuse large B-cell lymphoma: a comparison of IPI, R-IPI, and NCCN-IPI. Blood.

[11] Chorti A, Bangeas P, Papavramidis TS, Tsoulfas G (2018). Role of MicroRNA in the diagnosis and therapy of hepatic metastases from colorectal cancer. Microrna.

[12] Verter E, Berger Y, Perl G, Peretz I, Tovar A, Morgenstern S, Brenner B, Benchimol D, Kashtan H, Sadot E (2021). Neutrophil-to-lymphocyte ratio predicts recurrence pattern in patients with resectable colorectal liver metastases. Ann Surg Oncol.

[13] Zaitsu J, Yamashita Y, Ishikawa A, Saito A, Kagimoto A, Mimura T, Hirakawa T, Mito M, Fukuhara K, Senoo T (2021). Systemic inflammatory score predicts response and prognosis in patients with lung cancer treated with immunotherapy. Anticancer Res.

[14] Kasprzak A (2021). The role of tumor microenvironment cells in colorectal cancer (CRC) cachexia. Int J Mol Sci.

[15] Dolan RD, McSorley ST, Park JH, Watt DG, Roxburgh CS, Horgan PG, McMillan DC (2018). The prognostic value of systemic inflammation in patients undergoing surgery for colon cancer: comparison of composite ratios and cumulative scores. Brit J Cancer.

[16] Liao R, Peng C, Li M, Li DW, Jiang N, Li PZ, Ding X, Wu Q, Du CY, Gong JP (2018). Comparison and validation of the prognostic value of preoperative systemic immune cells in hepatocellular carcinoma after curative hepatectomy. Cancer Med-Us.

[17] Jagadesham VP, Lagarde SM, Immanuel A, Griffin SM (2017). Systemic inflammatory markers and outcome in patients with locally advanced adenocarcinoma of the oesophagus and gastro-oesophageal junction. Brit J Surg.

[18] Liu J, Li S, Zhang S, Liu Y, Ma L, Zhu J, Xin Y, Wang Y, Yang C, Cheng Y (2019). Systemic immune-inflammation index, neutrophil-to-lymphocyte ratio, platelet-to-lymphocyte ratio can predict clinical outcomes in patients with metastatic non-small-cell lung cancer treated with nivolumab. J Clin Lab Anal.

[19] Kusumanto YH, Dam WA, Hospers GA, Meijer C, Mulder NH (2003). Platelets and granulocytes, in particular the neutrophils, form important compartments for circulating vascular endothelial growth factor. Angiogenesis.

[20] Wang E, Shibutani M, Nagahara H, Fukuoka T, Iseki Y, Okazaki Y, Kashiwagi S, Tanaka H, Maeda K (2021). Prognostic value of the density of tumor-infiltrating lymphocytes in colorectal cancer liver metastases. Oncol Lett.

[21] Hamada T, Ishizaki H, Haruyama Y, Hamada R, Yano K, Kondo K, Kataoka H, Nanashima A (2020). Neutrophil-to-lymphocyte ratio and intratumoral CD45RO-positive T cells as predictive factors for longer survival of patients with colorectal liver metastasis after Hepatectomy. Tohoku J Exp Med.

[22] Moher D, Shamseer L, Clarke M, Ghersi D, Liberati A, Petticrew M, Shekelle P, Stewart LA (2015). Preferred reporting items for systematic review and meta-analysis protocols (PRISMA-P) 2015 statement. Syst Rev.

[23] Tierney JF, Stewart LA, Ghersi D, Burdett S, Sydes MR (2007). Practical methods for incorporating summary time-to-event data into meta-analysis. Trials.

[24] Wang Y, Liu Z, Xu D, Liu M, Wang K, Xing B (2019). Fibrinogen-albumin ratio index (FARI): a more promising inflammation-based prognostic marker for patients undergoing Hepatectomy for colorectal liver metastases. Ann Surg Oncol.

[25] Neal CP, Cairns V, Jones MJ, Masood MM, Nana GR, Mann CD, Garcea G, Dennison AR (2015). Prognostic performance of inflammation-based prognostic indices in patients with resectable colorectal liver metastases. Med Oncol.

[26] Neal CP, Mann CD, Garcea G, Briggs CD, Dennison AR, Berry DP (2011). Preoperative systemic inflammation and infectious complications after resection of colorectal liver metastases. Arch Surg.

[27] Neal CP, Mann CD, Sutton CD, Garcea G, Ong SL, Steward WP, Dennison AR, Berry DP (2009). Evaluation of the prognostic value of systemic inflammation and socioeconomic deprivation in patients with resectable colorectal liver metastases. Eur J Cancer.

[28] Mao R, Zhao JJ, Bi XY, Zhang YF, Li ZY, Huang Z, Zhou JG, Zhao H, Cai JQ (2019). A low neutrophil to lymphocyte ratio before preoperative chemotherapy predicts good outcomes after the resection of colorectal liver metastases. J Gastrointest Surg.

[29] Halazun KJ, Aldoori A, Malik HZ, Al-Mukhtar A, Prasad KR, Toogood GJ, Lodge JP (2008). Elevated preoperative neutrophil to lymphocyte ratio predicts survival following hepatic resection for colorectal liver metastases. Ejso-Eur J Surg Onc.

[30] Erstad DJ, Taylor MS, Qadan M, Axtell AL, Fuchs BC, Berger DL, Clancy TE, Tanabe KK, Chang DC, Ferrone CR (2020). Platelet and neutrophil to lymphocyte ratios predict survival in patients with resectable colorectal liver metastases. Am J Surg.

[31] Neofytou K, Smyth EC, Giakoustidis A, Khan AZ, Cunningham D, Mudan S (2014). Elevated platelet to lymphocyte ratio predicts poor prognosis after hepatectomy for liver-only colorectal metastases, and it is superior to neutrophil to lymphocyte ratio as an adverse prognostic factor. Med Oncol.

[32] Peng J, Li H, Ou Q, Lin J, Wu X, Lu Z, Yuan Y, Wan D, Fang Y, Pan Z (2017). Preoperative lymphocyte-to-monocyte ratio represents a superior predictor compared with neutrophil-to-lymphocyte and platelet-to-lymphocyte ratios for colorectal liver-only metastases survival. Oncotargets Ther.

[33] Zeman M, Maciejewski A, Poltorak S, Kryj M (2013). Evaluation of outcomes and treatment safety of patients with metastatic colorectal cancer to the liver with estimation of prognostic factors. Pol J Surg.

[34] Giakoustidis A, Neofytou K, Khan AZ, Mudan S (2015). Neutrophil to lymphocyte ratio predicts pattern of recurrence in patients undergoing liver resection for colorectal liver metastasis and thus the overall survival. J Surg Oncol.

[35] Wu Y, Li C, Zhao J, Yang L, Liu F, Zheng H, Wang Z, Xu Y (2016). Neutrophil-to-lymphocyte and platelet-to-lymphocyte ratios predict chemotherapy outcomes and prognosis in patients with colorectal cancer and synchronous liver metastasis. World J Surg Oncol.

[36] Zhang Y, Peng Z, Chen M, Liu F, Huang J, Xu L, Zhang Y, Chen M (2012). Elevated neutrophil to lymphocyte ratio might predict poor prognosis for colorectal liver metastasis after percutaneous radiofrequency ablation. Int J Hyperther.

[37] Weiner AA, Gui B, Newman NB, Nosher JL, Yousseff F, Lu SE, Foltz GM, Carpizo D, Lowenthal J, Zuckerman DA (2018). Predictors of Survival after Yttrium-90 radioembolization for colorectal cancer liver metastases. J Vasc Interv Radiol.

[38] Tohme S, Sukato D, Chalhoub D, McDonald KACA, Zajko A, Amesur N, Orons P, Marsh JW, Geller DA, Tsung A (2015). Neutrophil-lymphocyte ratio is a simple and novel biomarker for prediction of survival after radioembolization for metastatic colorectal cancer. Ann Surg Oncol.

[39] Zhang X, Jiang Y, Wang Y, Wang Z, Zhao L, Xue X, Sang S, Zhang L (2018). Prognostic role of neutrophil-lymphocyte ratio in esophageal cancer: a systematic review and meta-analysis. Medicine.

[40] Kamarajah SK, Marson EJ, Zhou D, Wyn-Griffiths F, Lin A, Evans R, Bundred JR, Singh P, Griffiths EA (2020). Meta-analysis of prognostic factors of overall survival in patients undergoing oesophagectomy for oesophageal cancer. Dis Esophagus.

[41] Szor DJ, Dias AR, Pereira MA, Ramos M, Zilberstein B, Cecconello I, Ribeiro-Junior U (2018). Prognostic role of neutrophil/lymphocyte ratio in resected gastric cancer: a systematic review and meta-analysis. Clinics.

[42] Mellor KL, Powell A, Lewis WG (2018). Systematic Review and Meta-Analysis of the Prognostic Significance of Neutrophil-Lymphocyte Ratio (NLR) After R0 Gastrectomy for Cancer. J Gastrointest Canc.

[43] Kotecha K, Singla A, Townend P, Merrett N (2022). Association between neutrophil-lymphocyte ratio and lymph node metastasis in gastric cancer: a meta-analysis. Medicine.

[44] Min GT, Li YM, Yao N, Wang J, Wang HP, Chen W (2018). The pretreatment neutrophil-lymphocyte ratio may predict prognosis of patients with liver cancer: a systematic review and meta-analysis. Clin Transplant.

[45] Wang Y, Peng C, Cheng Z, Wang X, Wu L, Li J, Huang C, Guo Q, Cai H (2018). The prognostic significance of preoperative neutrophil-lymphocyte ratio in patients with hepatocellular carcinoma receiving hepatectomy: a systematic review and meta-analysis. Int J Surg.

[46] Zeng F, Chen B, Zeng J, Wang Z, Xiao L, Deng G (2019). Preoperative neutrophil-lymphocyte ratio predicts the risk of microvascular invasion in hepatocellular carcinoma: a meta-analysis. Int J Biol Marker.

[47] Liu D, Heij LR, Czigany Z, Dahl E, Dulk MD, Lang SA, Ulmer TF, Neumann UP, Bednarsch J (2022). The prognostic value of neutrophil-to-lymphocyte ratio in cholangiocarcinoma: a systematic review and meta-analysis. Sci Rep-Uk.

[48] Naszai M, Kurjan A, Maughan TS (2021). The prognostic utility of pre-treatment neutrophil-to-lymphocyte-ratio (NLR) in colorectal cancer: a systematic review and meta-analysis. Cancer Med-Us.

[49] Hamid H, Davis GN, Trejo-Avila M, Igwe PO, Garcia-Marin A (2021). Prognostic and predictive value of neutrophil-to-lymphocyte ratio after curative rectal cancer resection: a systematic review and meta-analysis. Surg Oncol.

[50] Li H, Zhao Y, Zheng F (2019). Prognostic significance of elevated preoperative neutrophil-to-lymphocyte ratio for patients with colorectal cancer undergoing curative surgery: a meta-analysis. Medicine.

[51] Zhang N, Jiang J, Tang S, Sun G (2020). Predictive value of neutrophil-lymphocyte ratio and platelet-lymphocyte ratio in non-small cell lung cancer patients treated with immune checkpoint inhibitors: a meta-analysis. Int Immunopharmacol.

[52] Huang L, Jiang S, Shi Y (2022). Prognostic significance of baseline neutrophil-lymphocyte ratio in patients with non-small-cell lung cancer: a pooled analysis of open phase III clinical trial data. Future Oncol.

[53] Duan J, Pan L, Yang M (2018). Preoperative elevated neutrophil-to-lymphocyte ratio (NLR) and derived NLR are associated with poor prognosis in patients with breast cancer: a meta-analysis. Medicine.

[54] Yin X, Wu L, Yang H, Yang H (2019). Prognostic significance of neutrophil-lymphocyte ratio (NLR) in patients with ovarian cancer: a systematic review and meta-analysis. Medicine.

[55] Nunno VD, Mollica V, Gatto L, Santoni M, Cosmai L, Porta C, Massari F (2019). Prognostic impact of neutrophil-to-lymphocyte ratio in renal cell carcinoma: a systematic review and meta-analysis. Immunotherapy.

[56] Shao Y, Wu B, Jia W, Zhang Z, Chen Q, Wang D (2020). Prognostic value of pretreatment neutrophil-to-lymphocyte ratio in renal cell carcinoma: a systematic review and meta-analysis. Bmc Urol.

[57] Tang L, Li X, Wang B, Luo G, Gu L, Chen L, Liu K, Gao Y, Zhang X (2016). Prognostic value of neutrophil-to-lymphocyte ratio in localized and advanced prostate cancer: a systematic review and meta-analysis. PLoS ONE.

[58] Arneth B (2019). Tumor microenvironment. Medicina-Lithuania.

[59] Hinshaw DC, Shevde LA (2019). The tumor microenvironment innately modulates cancer progression. Cancer Res.

[60] Anderson NM, Simon MC (2020). The tumor microenvironment. Curr Biol.

[61] Mantovani A (2009). Cancer: inflaming metastasis. Nature.

[62] Singh N, Baby D, Rajguru JP, Patil PB, Thakkannavar SS, Pujari VB (2019). Inflammation and cancer. Ann Afr Med.

[63] Chmielewski PP, Strzelec B (2018). Elevated leukocyte count as a harbinger of systemic inflammation, disease progression, and poor prognosis: a review. Folia Morphol.

[64] Kraus RF, Gruber MA (2021). Neutrophils-from bone marrow to first-line defense of the innate immune system. Front Immunol.

[65] Mizuno R, Kawada K, Itatani Y, Ogawa R, Kiyasu Y, Sakai Y (2019). The role of tumor-associated neutrophils in colorectal cancer. Int J Mol Sci.

[66] Silzle T, Randolph GJ, Kreutz M, Kunz-Schughart LA (2004). The fibroblast: sentinel cell and local immune modulator in tumor tissue. Int J Cancer.

[67] Elebiyo TC, Rotimi D, Evbuomwan IO, Maimako RF, Iyobhebhe M, Ojo OA, Oluba OM, Adeyemi OS (2022). Reassessing vascular endothelial growth factor (VEGF) in anti-angiogenic cancer therapy. Cancer Treat Res Commun.

[68] Kessenbrock K, Plaks V, Werb Z (2010). Matrix metalloproteinases: regulators of the tumor microenvironment. Cell.

[69] Gregory AD, Houghton AM (2011). Tumor-associated neutrophils: new targets for cancer therapy. Cancer Res.

[70] Ebrahem Q, Chaurasia SS, Vasanji A, Qi JH, Klenotic PA, Cutler A, Asosingh K, Erzurum S, Anand-Apte B (2010). Cross-talk between vascular endothelial growth factor and matrix metalloproteinases in the induction of neovascularization in vivo. Am J Pathol.

[71] Belaaouaj A, McCarthy R, Baumann M, Gao Z, Ley TJ, Abraham SN, Shapiro SD (1998). Mice lacking neutrophil elastase reveal impaired host defense against gram negative bacterial sepsis. Nat Med.

[72] Weinrauch Y, Drujan D, Shapiro SD, Weiss J, Zychlinsky A (2002). Neutrophil elastase targets virulence factors of enterobacteria. Nature.

[73] Houghton AM, Rzymkiewicz DM, Ji H, Gregory AD, Egea EE, Metz HE, Stolz DB, Land SR, Marconcini LA, Kliment CR (2010). Neutrophil elastase-mediated degradation of IRS-1 accelerates lung tumor growth. Nat Med.

[74] Dirican N, Karakaya YA, Gunes S, Daloglu FT, Dirican A (2017). Association of intra-tumoral tumour-infiltrating lymphocytes and neutrophil-to-lymphocyte ratio is an independent prognostic factor in non-small cell lung cancer. Clin Respir J.

[75] Mlecnik B, Van den Eynde M, Bindea G, Church SE, Vasaturo A, Fredriksen T, Lafontaine L, Haicheur N, Marliot F, Debetancourt D (2018). Comprehensive intrametastatic immune quantification and major impact of immunoscore on survival. Jnci-J Natl Cancer I.

[76] Chen ZY, Raghav K, Lieu CH, Jiang ZQ, Eng C, Vauthey JN, Chang GJ, Qiao W, Morris J, Hong D (2015). Cytokine profile and prognostic significance of high neutrophil-lymphocyte ratio in colorectal cancer. Brit J Cancer.

[77] Itoh S, Yoshizumi T, Yugawa K, Imai D, Yoshiya S, Takeishi K, Toshima T, Harada N, Ikegami T, Soejima Y (2020). Impact of immune response on outcomes in hepatocellular carcinoma: association with vascular formation. Hepatology.

[78] Fuchs TL, Sioson L, Sheen A, Jafari-Nejad K, Renaud CJ, Andrici J, Ahadi M, Chou A, Gill AJ (2020). Assessment of tumor-infiltrating lymphocytes using international TILs working group (ITWG) system is a strong predictor of overall survival in colorectal carcinoma: a study of 1034 patients. Am J Surg Pathol.

[79] Ceprnja T, Mrklic I, Peric BM, Marusic Z, Blazicevic V, Spagnoli GC, Juretic A, Capkun V, Tecic VA, Vrdoljak E (2022). Prognostic significance of lymphocyte infiltrate localization in triple-negative breast cancer. J Pers Med.

[80] Fridlender ZG, Sun J, Kim S, Kapoor V, Cheng G, Ling L, Worthen GS, Albelda SM (2009). Polarization of tumor-associated neutrophil phenotype by TGF-beta: "N1" versus "N2" TAN. Cancer Cell.

