# Supplementary figures and images for: The prognostic utility of preoperative neutrophil-to-lymphocyte ratio (NLR) in patients with colorectal liver metastasis: a systematic review and meta-analysis
Source: Cancer Cell Int. 2023 Feb 28;23:39. doi: 10.1186/s12935-023-02876-z (PMC9976405; doi:10.1186/s12935-023-02876-z)

**Additional file**


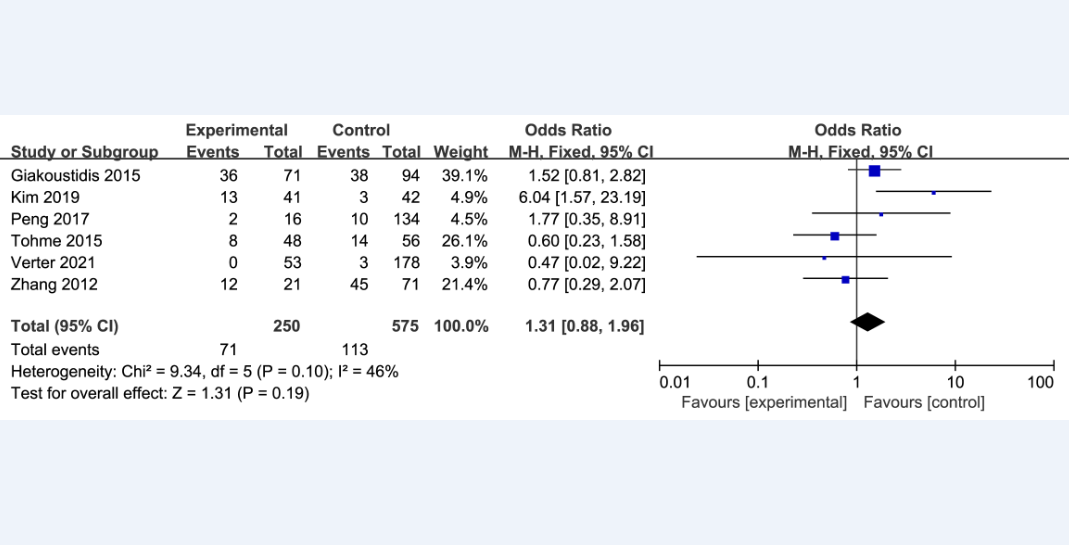


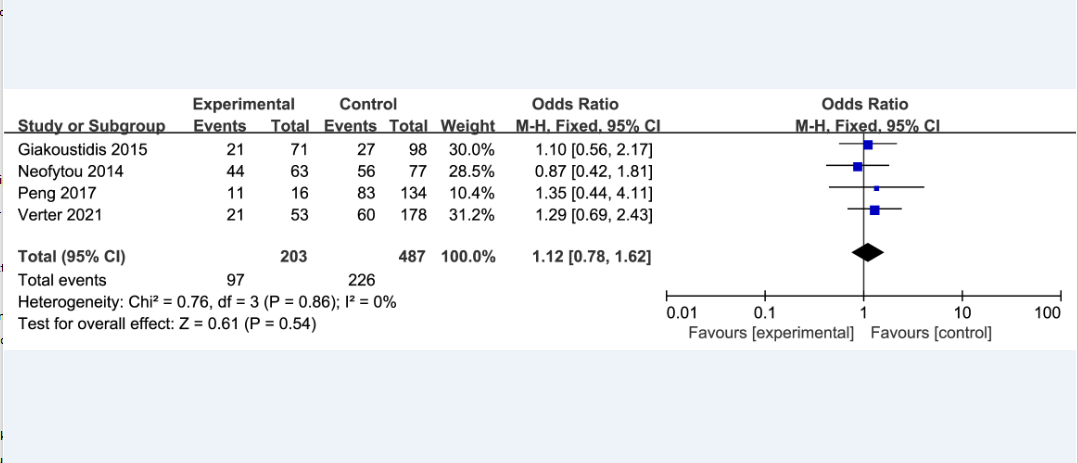


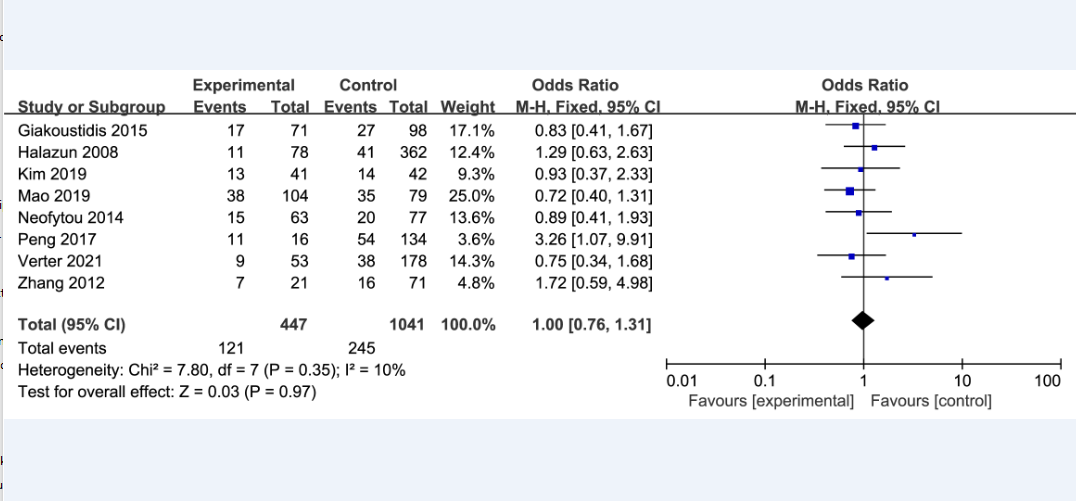


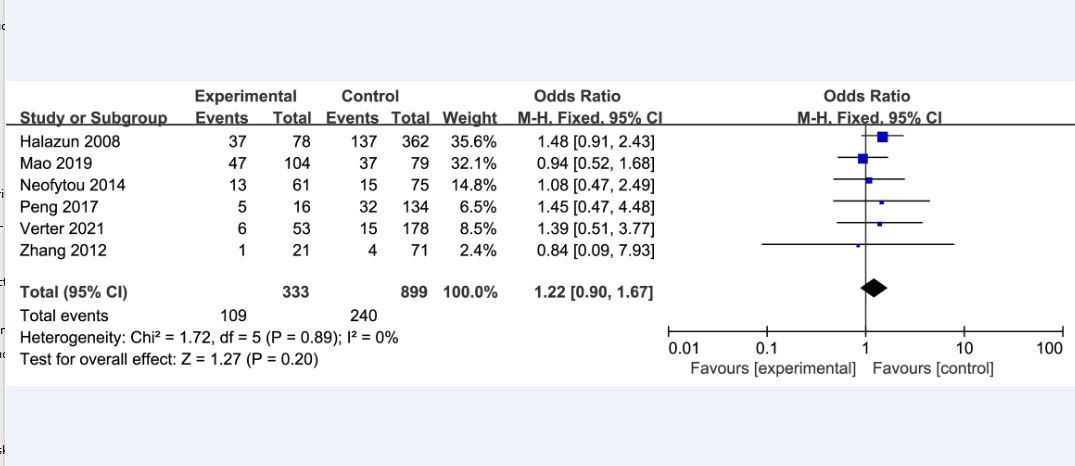


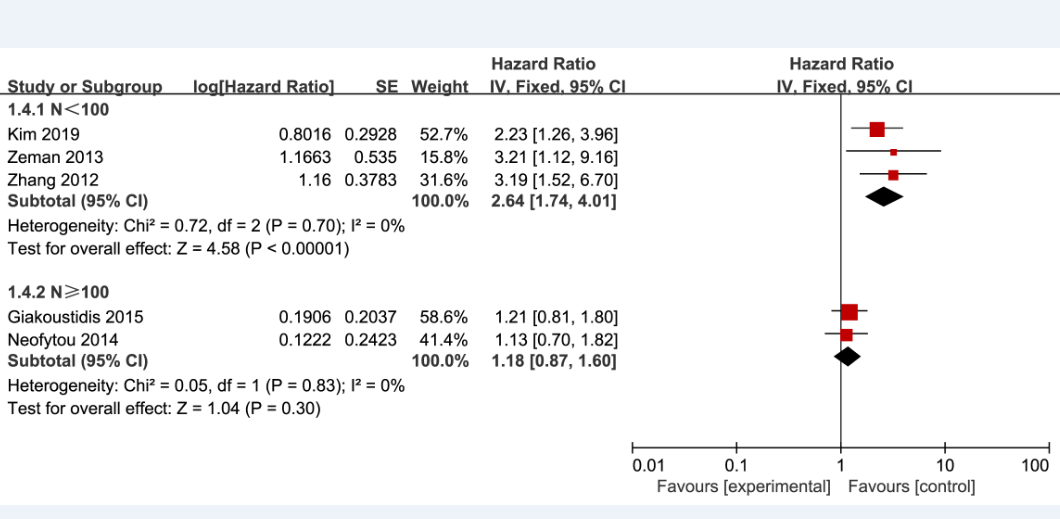


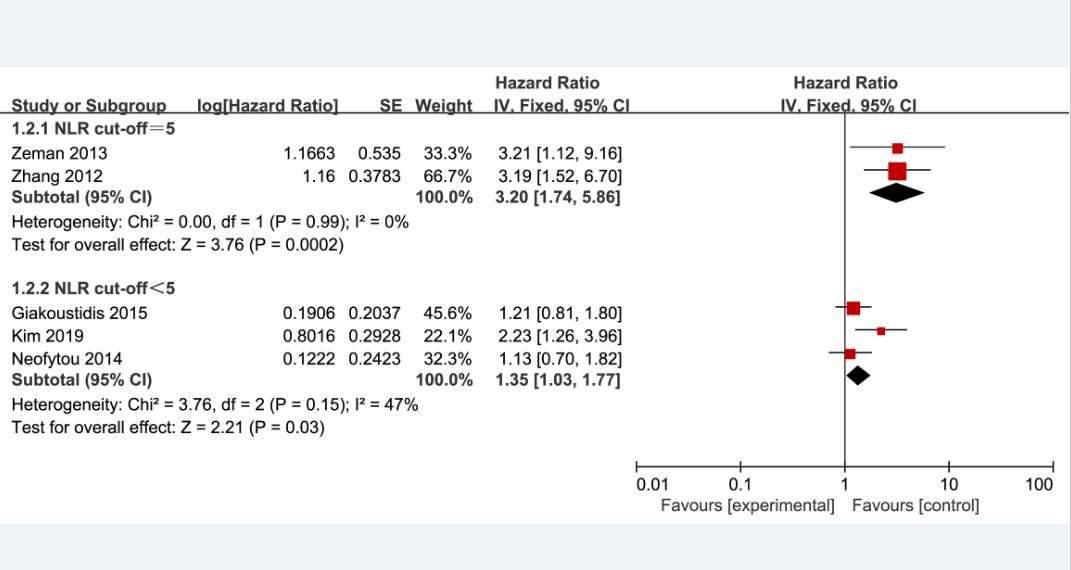


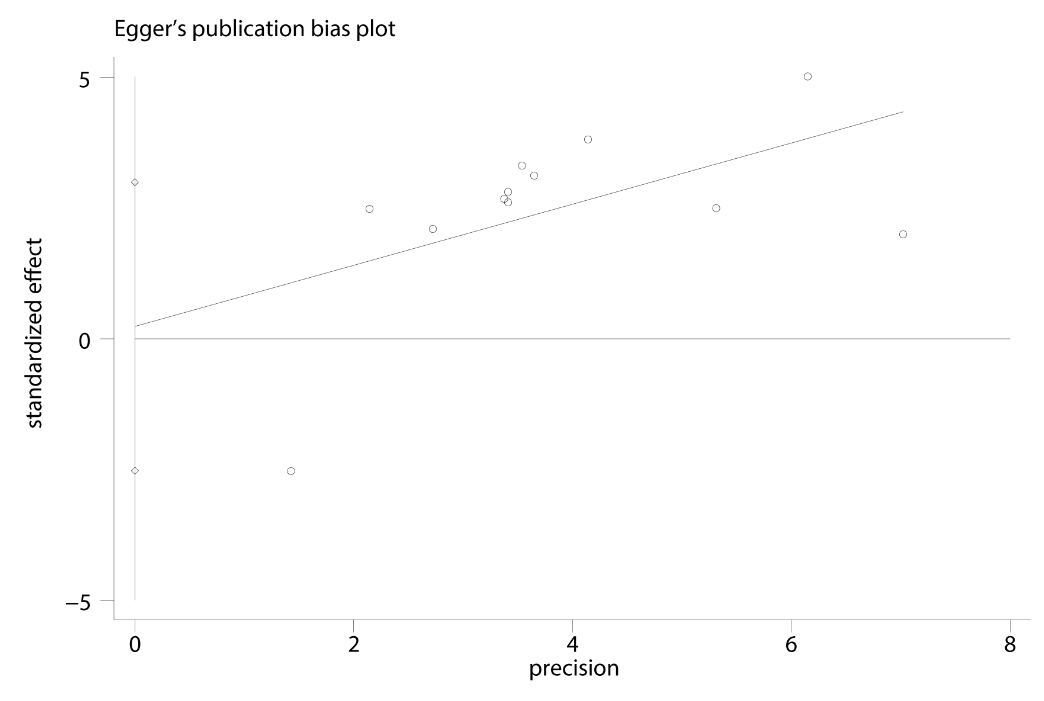


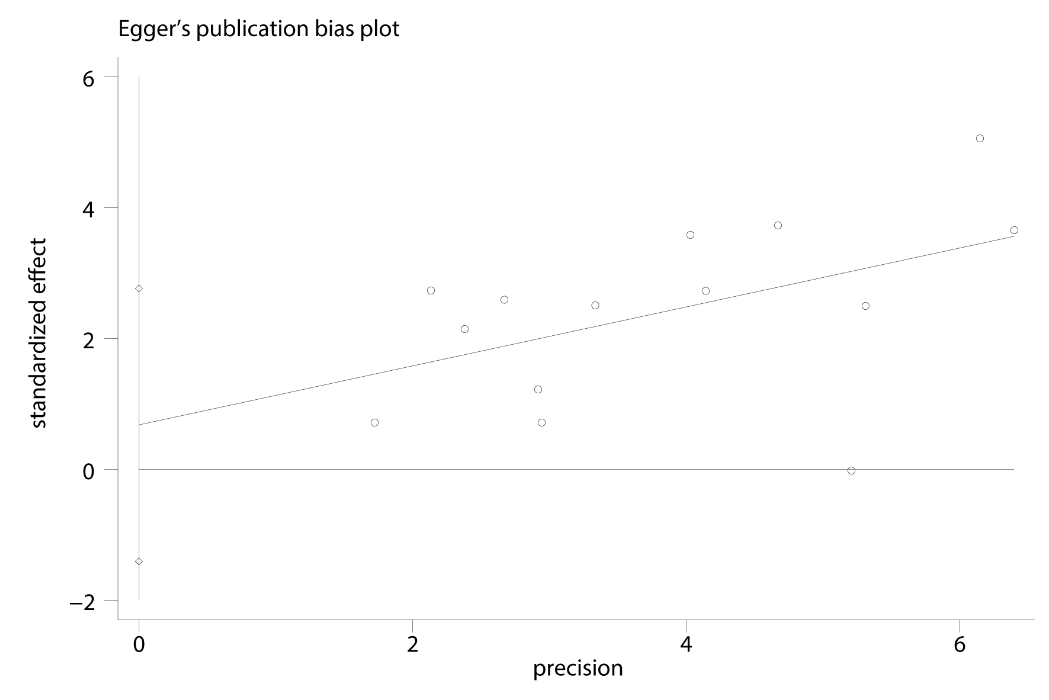


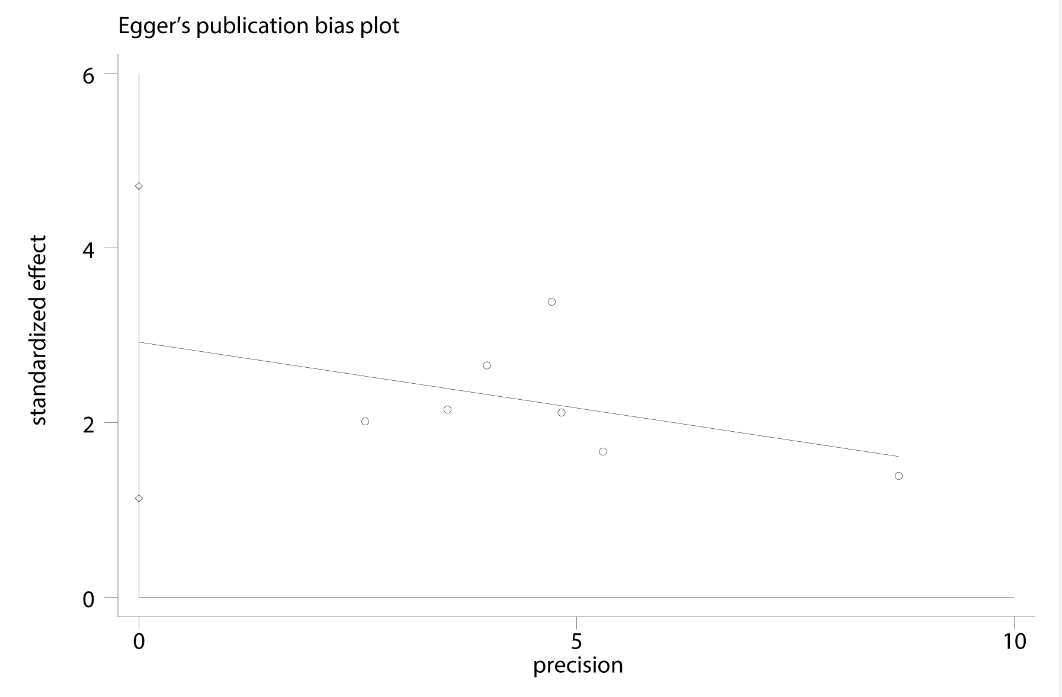


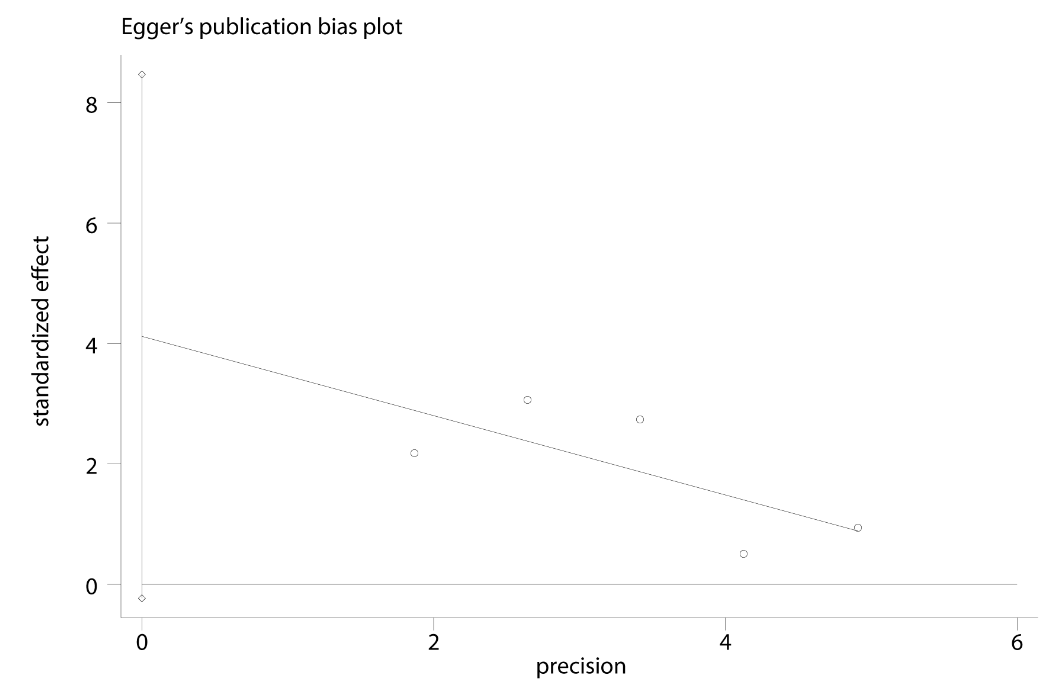


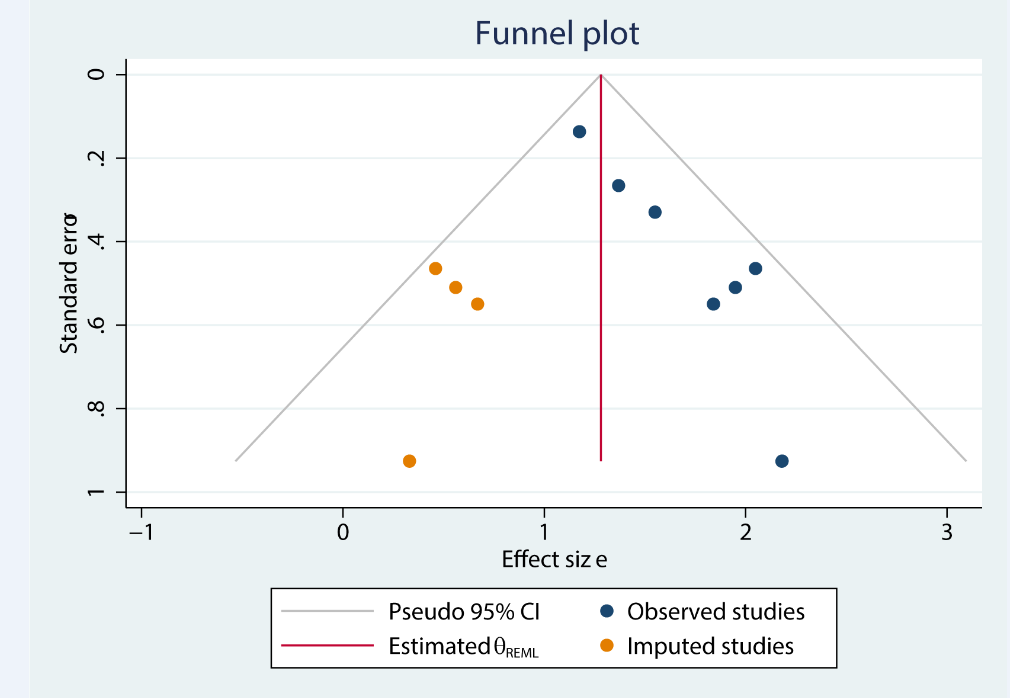

Supplement: Supplementary file 1 — Additional file 1: Figure S1. Forest plot of the relationship between preoperative NLR and Clinicopathological features in CRLM patients (a: Clinicopathological features; b: CEA; c: primary tumor size; d: time to metastasis). Figure S2. Forest plot for subgroup analysis of the correlation between NLR and multivariate DFS in CRLM patients (a: sample size; b: NLR cut-off value). Figure S3. Publication bias (a: Egger’s publication bias plot of univariate OS; b: Egger's publication bias plot of multivariate OS; c: Egger's publication bias plot of univariate DFS; d: Egger's publication bias plot of multivariate DFS; e: Funnel plot after univariate DFS application of trim-and-fill). [file 12935_2023_2876_MOESM1_ESM.docx]
