# Supplementary material for: The prognostic utility of preoperative neutrophil-to-lymphocyte ratio (NLR) in patients with colorectal liver metastasis: a systematic review and meta-analysis
Source: Cancer Cell Int. 2023 Feb 28;23:39. doi: 10.1186/s12935-023-02876-z (PMC9976405; doi:10.1186/s12935-023-02876-z)
Supplement: Supplementary file 2 — Additional file 2: Table S1. Search strategy. [file 12935_2023_2876_MOESM2_ESM.pdf]

Table S1. Search strategy

Pubmed:

#1	Colorectal Neoplasms [Mesh]
#2	Colorectal Neoplasm [Title/Abstract] OR Neoplasm, Colorectal [Title/Abstract] OR Neoplasms, Colorectal [Title/Abstract] OR Colorectal Tumors [Title/Abstract] OR Colorectal Tumor [Title/Abstract] OR Tumor, Colorectal [Title/Abstract] OR Tumors, Colorectal [Title/Abstract] OR Colorectal Cancer [Title/Abstract] OR Cancer, Colorectal [Title/Abstract] OR Cancers, Colorectal [Title/Abstract] OR Colorectal Cancers [Title/Abstract] OR Colorectal Carcinoma [Title/Abstract] OR Carcinoma, Colorectal [Title/Abstract] OR Carcinomas, Colorectal [Title/Abstract] OR Colorectal Carcinomas [Title/Abstract]
#3	#1 OR #2
#4	Colonic Neoplasms [Mesh]
#5	Colonic Neoplasm [Title/Abstract] OR Neoplasm, Colonic [Title/Abstract] OR Neoplasms, Colonic [Title/Abstract] OR Colon Neoplasms [Title/Abstract] OR Colon Neoplasm [Title/Abstract] OR Neoplasm, Colon [Title/Abstract] OR Neoplasms, Colon [Title/Abstract] OR Cancer of Colon [Title/Abstract] OR Colon Cancers [Title/Abstract] OR Colon Cancer [Title/Abstract] OR Cancer, Colon [Title/Abstract] OR Cancers, Colon [Title/Abstract] OR Cancer of the Colon [Title/Abstract] OR Colonic Cancer [Title/Abstract] OR Cancer, Colonic [Title/Abstract] OR Cancers, Colonic [Title/Abstract] OR Colonic Cancers [Title/Abstract] OR Colon Adenocarcinoma [Title/Abstract] OR Adenocarcinoma, Colon [Title/Abstract] OR Adenocarcinomas, Colon [Title/Abstract] OR Colon Adenocarcinomas [Title/Abstract]
#6	#4 OR #5
#7	Rectal Neoplasms [Title/Abstract]
#8	Neoplasm, Rectal [Title/Abstract] OR Rectal Neoplasm [Title/Abstract] OR Rectum Neoplasms [Title/Abstract] OR Neoplasm, Rectum [Title/Abstract] OR Rectum Neoplasm [Title/Abstract] OR Rectal Tumors [Title/Abstract] OR Rectal Tumor [Title/Abstract] OR Tumor, Rectal [Title/Abstract] OR Neoplasms, Rectal [Title/Abstract] OR Cancer of Rectum [Title/Abstract] OR Rectum Cancers [Title/Abstract] OR Rectal Cancer [Title/Abstract] OR Cancer, Rectal [Title/Abstract] OR Rectal Cancers [Title/Abstract] OR Rectum Cancer [Title/Abstract] OR Cancer, Rectum [Title/Abstract] OR Cancer of the Rectum [Title/Abstract]
#9	#7 OR #8
#10	#3 OR #6 OR #9
#11	Neoplasm Metastasis [Mesh]
#12	Neoplasm Metastases [Title/Abstract] OR Metastases, Neoplasm [Title/Abstract] OR Metastasis, Neoplasm [Title/Abstract] OR Metastases [Title/Abstract] OR Metastasis [Title/Abstract] OR Metastatic [Title/Abstract]
#13	#11 OR #12
#14	#10 AND #13
#15	Colorectal liver metastasis OR Colo-rectal liver metastases [Title/Abstract] OR Colo-rectal liver metastasis [Title/Abstract] OR Colorectal hepatic metastases [Title/Abstract] OR Colorectal hepatic metastasis [Title/Abstract] OR Colorectal liver

	metastases [Title/Abstract] OR Hepatic metastases from colorectal cancer [Title/Abstract] OR Hepatic metastases from colorectal carcinoma [Title/Abstract] OR Hepatic metastasis from colorectal cancer [Title/Abstract] OR Hepatic metastasis from colorectal carcinoma [Title/Abstract] OR CRLM [Title/Abstract]
#16	#14 OR #15
#17	Neutrophil-to-lymphocyte ratio OR Neutrophil lymphocyte ratio [Title/Abstract] OR Neutrophil-lymphocyte ratio [Title/Abstract] OR Neutrophil-lymphocyte [Title/Abstract] OR Neutrophil lymphocyte-ratio [Title/Abstract] OR NLR [Title/Abstract] OR Inflammatory markers [Title/Abstract]
#18	#16 AND #17

Embase:

#1. 'colorectal tumor'/exp
#2. 'colorectal neoplasm':ti,ab
#3. 'neoplasm, colorectal':ti,ab
#4. 'neoplasms, colorectal':ti,ab
#5. 'colorectal tumors':ti,ab
#6. 'tumor, colorectal':ti,ab
#7. 'tumors, colorectal':ti,ab
#8. 'colorectal cancer':ti,ab
#9. 'cancer, colorectal':ti,ab
#10. 'cancers, colorectal':ti,ab
#11. 'colorectal cancers':ti,ab
#12. 'colorectal carcinoma':ti,ab
#13. 'carcinoma, colorectal':ti,ab
#14. 'carcinomas, colorectal':ti,ab
#15. 'colorectal carcinomas':ti,ab
#16. 'colorectal neoplasms':ti,ab
#17. #1 OR #2 OR #3 OR #4 OR #5 OR #6 OR #7 OR #8 OR #9 OR #10 OR #11 OR #12 OR #13 OR #14 OR #15 OR #16
#18. 'colon tumor'/exp
#19. 'colonic neoplasms':ti,ab
#20. 'colonic neoplasm':ti,ab
#21. 'neoplasm, colonic':ti,ab
#22. 'neoplasms, colonic':ti,ab
#23. 'colon neoplasms':ti,ab
#24. 'colon neoplasm':ti,ab
#25. 'neoplasm, colon':ti,ab
#26. 'neoplasms, colon':ti,ab
#27. 'cancer of colon':ti,ab
#28. 'colon cancers':ti,ab
#29. 'colon cancer':ti,ab
#30. 'cancer, colon':ti,ab

#31. 'cancers, colon':ti,ab
#32. 'cancer of the colon':ti,ab
#33. 'colonic cancer':ti,ab
#34. 'cancer, colonic':ti,ab
#35. 'cancers, colonic':ti,ab
#36. 'colonic cancers':ti,ab
#37. 'colon adenocarcinoma':ti,ab
#38. 'adenocarcinoma, colon':ti,ab
#39. 'adenocarcinomas, colon':ti,ab
#40. 'colon adenocarcinomas':ti,ab
#41. #18 OR #19 OR #20 OR #21 OR #22 OR #23 OR #24 OR #25 OR #26 OR #27 OR #28 OR #29 OR #30 OR #31 OR #32 OR #33 OR #34 OR #35 OR #36 OR #37 OR #38 OR #39 OR #40
#42. 'rectum tumor'/exp
#43. 'neoplasm, rectal':ti,ab
#44. 'rectal neoplasm':ti,ab
#45. 'rectum neoplasms':ti,ab
#46. 'neoplasm, rectum':ti,ab
#47. 'rectum neoplasm':ti,ab
#48. 'rectal tumors':ti,ab
#49. 'rectal neoplasms':ti,ab
#50. 'tumor, rectal':ti,ab
#51. 'neoplasms, rectal':ti,ab
#52. 'cancer of rectum':ti,ab
#53. 'rectum cancers':ti,ab
#54. 'rectal cancer':ti,ab
#55. 'cancer, rectal':ti,ab
#56. 'rectal cancers':ti,ab
#57. 'rectum cancer':ti,ab
#58. 'cancer, rectum':ti,ab
#59. 'cancer of the rectum':ti,ab
#60. #42 OR #43 OR #44 OR #45 OR #46 OR #47 OR #48 OR #49 OR #50 OR #51 OR #52 OR #53 OR #54 OR #55 OR #56 OR #57 OR #58 OR #59
#61. #17 OR #41 OR #60
#62. 'metastasis'/exp
#63. 'metastasis':ti,ab
#64. 'metastases, neoplasm':ti,ab
#65. 'metastasis, neoplasm':ti,ab
#66. 'metastases':ti,ab
#67. 'neoplasm metastasis':ti,ab
#68. 'metastatic':ti,ab
#69. #62 OR #63 OR #64 OR #65 OR #66 OR #67 OR #68
#70. #60 AND #69

#71. 'colorectal liver metastasis'/exp
#72. 'colo-rectal liver metastases':ti,ab
#73. 'colo-rectal liver metastasis':ti,ab
#74. 'colorectal hepatic metastases':ti,ab
#75. 'colorectal hepatic metastasis':ti,ab
#76. 'colorectal liver metastases':ti,ab
#77. 'hepatic metastases from colorectal cancer':ti,ab
#78. 'hepatic metastases from colorectal carcinoma':ti,ab
#79. 'hepatic metastasis from colorectal cancer':ti,ab
#80. 'hepatic metastasis from colorectal carcinoma':ti,ab
#81. 'crlm':ti,ab
#82. #71 OR #72 OR #73 OR #74 OR #75 OR #76 OR #77 OR #78 OR #79 OR #80 OR #81
#83. #70 OR #82
#84. 'neutrophil lymphocyte ratio'/exp
#85. 'neutrophil-to-lymphocyte ratio':ti,ab
#86. 'neutrophil-lymphocyte ratio':ti,ab
#87. 'neutrophil-lymphocyte':ti,ab
#88. 'neutrophil lymphocyte-ratio':ti,ab
#89. 'nlr':ti,ab
#90. 'inflammatory markers':ti,ab
#91. #84 OR #85 OR #86 OR #87 OR #88 OR #89 OR #90
#92. #83 AND #91

Cochrane Library:

#1	MeSH descriptor: [Colorectal Neoplasms] explode all trees
#2	(Colorectal Neoplasm):ti,ab,kw OR (Neoplasm, Colorectal):ti,ab,kw OR (Neoplasms, Colorectal):ti,ab,kw OR (Colorectal Tumors):ti,ab,kw OR (Colorectal Tumor):ti,ab,kw (Word variations have been searched)
#3	(Tumor, Colorectal):ti,ab,kw OR (Tumors, Colorectal):ti,ab,kw OR (Colorectal Cancer):ti,ab,kw OR (Cancer, Colorectal):ti,ab,kw OR (Cancers, Colorectal):ti,ab,kw (Word variations have been searched)
#4	(Colorectal Cancers):ti,ab,kw OR (Colorectal Carcinoma):ti,ab,kw OR (Carcinoma, Colorectal):ti,ab,kw OR (Carcinomas, Colorectal):ti,ab,kw OR (Colorectal Carcinomas):ti,ab,kw (Word variations have been searched)
#5	#1 or #2 or #3 or #4
#6	MeSH descriptor: [Colonic Neoplasms] explode all trees
#7	(Colonic Neoplasm):ti,ab,kw OR (Neoplasm, Colonic):ti,ab,kw OR (Neoplasms, Colonic):ti,ab,kw OR (Colon Neoplasms):ti,ab,kw OR (Colon Neoplasm):ti,ab,kw (Word variations have been searched)
#8	(Neoplasm, Colon):ti,ab,kw OR (Neoplasms, Colon):ti,ab,kw OR (Cancer of Colon):ti,ab,kw OR (Colon Cancers):ti,ab,kw OR (Colon Cancer):ti,ab,kw (Word variations have been searched)
#9	(Cancer, Colon):ti,ab,kw OR (Cancers, Colon):ti,ab,kw OR (Cancer of the

	Colon):ti,ab,kw OR (Colonic Cancer):ti,ab,kw OR (Cancer, Colonic):ti,ab,kw (Word variations have been searched)
#10	(Cancers, Colonic):ti,ab,kw OR (Colonic Cancers):ti,ab,kw OR (Colon Adenocarcinoma):ti,ab,kw OR (Adenocarcinoma, Colon):ti,ab,kw OR (Adenocarcinomas, Colon):ti,ab,kw (Word variations have been searched)
#11	(Colon Adenocarcinomas):ti,ab,kw (Word variations have been searched)
#12	#6 or #7 or #8 or #9 or #10 or #11
#13	MeSH descriptor: [Rectal Neoplasms] explode all trees
#14	(Neoplasm, Rectal):ti,ab,kw OR (Rectal Neoplasm):ti,ab,kw OR (Rectum Neoplasms):ti,ab,kw OR (Neoplasm, Rectum):ti,ab,kw OR (Rectum Neoplasm):ti,ab,kw (Word variations have been searched)
#15	(Rectal Tumors):ti,ab,kw OR (Rectal Tumor):ti,ab,kw OR (Tumor, Rectal):ti,ab,kw OR (Neoplasms, Rectal):ti,ab,kw OR (Cancer of Rectum):ti,ab,kw (Word variations have been searched)
#16	(Rectum Cancers):ti,ab,kw OR (Rectal Cancer):ti,ab,kw OR (Cancer, Rectal):ti,ab,kw OR (Rectal Cancers):ti,ab,kw OR (Rectum Cancer):ti,ab,kw (Word variations have been searched)
#17	(Cancer, Rectum):ti,ab,kw OR (Cancer of the Rectum):ti,ab,kw (Word variations have been searched)
#18	#13 or #14 or #15 or #16 or #17
#19	#5 or #12 or #18
#20	MeSH descriptor: [Neoplasm Metastasis] explode all trees
#21	(Neoplasm Metastases):ti,ab,kw OR (Metastases, Neoplasm):ti,ab,kw OR (Metastasis, Neoplasm):ti,ab,kw OR (Metastases):ti,ab,kw OR (Metastasis):ti,ab,kw (Word variations have been searched)
#22	(Metastatic):ti,ab,kw (Word variations have been searched)
#23	#20 or #21 or #22
#24	#19 and #23
#25	(Colorectal liver metastasis):ti,ab,kw OR (Colo-rectal liver metastases):ti,ab,kw OR (Colo-rectal liver metastasis):ti,ab,kw OR (Colorectal hepatic metastases):ti,ab,kw OR (Colorectal hepatic metastasis):ti,ab,kw (Word variations have been searched)
#25	(Colorectal liver metastasis):ti,ab,kw OR (Colo-rectal liver metastases):ti,ab,kw OR (Colo-rectal liver metastasis):ti,ab,kw OR (Colorectal hepatic metastases):ti,ab,kw OR (Colorectal hepatic metastasis):ti,ab,kw (Word variations have been searched)
#27	(CRLM):ti,ab,kw (Word variations have been searched)
#28	#25 or #26 or #27
#29	#24 or #28
#30	(Neutrophil-to-lymphocyte ratio):ti,ab,kw OR (Neutrophil lymphocyte ratio):ti,ab,kw OR (Neutrophil-lymphocyte ratio):ti,ab,kw OR (Neutrophil-lymphocyte):ti,ab,kw OR (Neutrophil lymphocyte-ratio):ti,ab,kw (Word variations have been searched)
#31	(NLR):ti,ab,kw OR (Inflammatory markers):ti,ab,kw (Word variations have been searched)
#32	#30 or #31

#33	#29 and #32

Web of Science:

1: TS=(Colorectal Neoplasm* OR Colorectal Neoplasm OR Neoplasm, Colorectal OR Neoplasm*, Colorectal OR Colorectal Tumor* OR Colorectal Tumor OR Tumor, Colorectal OR Tumor*, Colorectal OR Colorectal Cancer OR Cancer, Colorectal OR Cancer*, Colorectal OR Colorectal Cancer* OR Colorectal Carcinoma OR Carcinoma, Colorectal OR Carcinoma*, Colorectal OR Colorectal Carcinoma*)
2: TS=(Colonic Neoplasm* OR Colonic Neoplasm OR Neoplasm, Colonic OR Neoplasm*, Colonic OR Colon Neoplasm* OR Colon Neoplasm OR Neoplasm, Colon OR Neoplasm*, Colon OR Cancer of Colon OR Colon Cancer* OR Colon Cancer OR Cancer, Colon OR Cancer*, Colon OR Cancer of the Colon OR Colonic Cancer OR Cancer, Colonic OR Cancer*, Colonic OR Colonic Cancer* OR Colon Adenocarcinoma OR Adenocarcinoma, Colon OR Adenocarcinoma*, Colon OR Colon Adenocarcinoma*)
3: TS=(Rectal Neoplasm* OR Neoplasm, Rectal OR Rectal Neoplasm OR Rectum Neoplasm* OR Neoplasm, Rectum OR Rectum Neoplasm OR Rectal Tumor* OR Rectal Tumor OR Tumor, Rectal OR Neoplasm*, Rectal OR Cancer of Rectum OR Rectum Cancer* OR Rectal Cancer OR Cancer, Rectal OR Rectal Cancer* OR Rectum Cancer OR Cancer, Rectum OR Cancer of the Rectum)
4: #1 OR #2 OR #3
5: TS=(Neoplasm Metastasis OR Neoplasm Metastases OR Metastases, Neoplasm OR Metastasis, Neoplasm OR Metastases OR Metastasis OR Metastatic)
6: TS=(Liver OR Liver* OR Hepatopathy)
7: #6 AND #5 AND #4
8: TS=(Colorectal liver metastasis OR Colo-rectal liver metastases OR Colo-rectal liver metastasis OR Colorectal hepatic metastases OR Colorectal hepatic metastasis OR Colorectal liver metastases OR Hepatic metastases from colorectal cancer OR Hepatic metastases from colorectal carcinoma OR Hepatic metastasis from colorectal cancer OR Hepatic metastasis from colorectal carcinoma OR CRLM)
9: #8 OR #7
10: TS=(Neutrophil-to-lymphocyte ratio OR Neutrophil lymphocyte ratio OR Neutrophil-lymphocyte ratio OR Neutrophil-lymphocyte OR Neutrophil lymphocyte-ratio OR NLR OR Inflammatory marker*)
11: #10 AND #9
